# Canagliflozin attenuates Parkinson’s disease and is associated with modulation of gut-inflammasome–brain axis in rats

**DOI:** 10.1007/s10787-026-02292-5

**Published:** 2026-06-22

**Authors:** Nada K. Gamal, Rafik Fakhry, Youmna Hatem, Engy Rashed, Reem Marzouk, Ahmed K. M. Bukr, Kerolos Safwat, Mohamed Mamdouh, Ahmed AbdElFatah, Abdelrahman Atallah, Heba Attia, Iriny M. Ayoub, Mina Y. George

**Affiliations:** 1 Department of Pharmacology and Toxicology, Faculty of Pharmacy, Ain Shams University, Cairo, 11566 Egypt; 2 PharmD Clinical Program, Faculty of Pharmacy, Ain Shams University, Cairo, 11566 Egypt; 3 Department of Microbiology and Immunology, Faculty of Pharmacy, Cairo University, Cairo, 11562 Egypt; 4 Department of Pharmacognosy, Faculty of Pharmacy, Ain Shams University, Abbassiya, Cairo, 11566 Egypt; 5 Biology Department, School of Pharmacy, Newgiza University, Giza, Egypt

**Keywords:** Parkinson’s disease, Canagliflozin, Gut–brain axis, Oxidative stress, NLRP3 inflammasome, α-synuclein

## Abstract

Recent evidence links gut dysbiosis and the gut-brain axis to the pathophysiology of Parkinson’s disease (PD), which is considered one of the most common neurodegenerative illnesses and is characterized by motor symptoms and a gradual loss of nigral dopaminergic neurons. Canagliflozin (CANA), a sodium–glucose cotransporter-2 inhibitor with emerging anti-inflammatory and antioxidant properties, has recently gained attention for its neuroprotective effects beyond glycemic control. This research aimed to examine the neuroprotective potential of CANA in a rat model of PD, focusing on its potential effect on the gut-inflammasome–brain axis. Rats were treated with rotenone (ROT) (2 mg/kg, subcutaneous) and/or CANA (20 mg/kg, oral) for 30 consecutive days. Behavioral assessment (*n* = 13), biochemical assays (*n* = 6), and histological and immunohistochemical analysis (*n* = 3) were performed. Microbiome profiling (*n* = 3) using 16S rRNA amplicon sequencing was conducted using three pooled fecal samples/group, with each pool prepared from fecal material collected from three rats and fecal metabolomic profiling (*n* = 5) was performed to assess microbial metabolites. ROT intoxication caused significant motor impairments, including decreased locomotor activity, poor coordination, and increased catalepsy. Further histological examination demonstrated dopaminergic neuronal loss and α-synuclein aggregation in the substantia nigra and striatum. CANA treatment enhanced motor function, conserved neuronal integrity, and decreased α-synuclein accumulation. Moreover, ROT altered the gut microbiota with selective taxonomic shifts, including enriched abundance of *Parabacteroides* and *Ruminococcaceae* with depleted *Prevotella*-related taxa, in addition to fecal metabolite profile alteration. Consequently, it increased colonic oxidative stress and weakened tight junctions, all of which enhanced LPS translocation. This systemic endotoxemia generated oxidative stress, dyslipidemia, and NF-κB/NLRP3 inflammasome activation. Conversely, CANA restored gut microbial balance and their metabolites, and gut integrity, decreased LPS leakage, and reduced systemic and central oxidative stress and inflammation, preventing inflammasome activation and α-synuclein aggregation. Collectively, these findings suggest that CANA may exert neuroprotective effects in ROT-induced PD associated with modulating the gut-inflammasome–brain axis.

**Supplementary Information:**

The online version contains supplementary material available at 10.1007/s10787-026-02292-5.

## Introduction

Parkinson’s disease (PD) is one of the most common neurodegenerative diseases globally, which mainly affects elderly people, with a worldwide prevalence estimated at 200–400 per 100,000 in high-income nations (Grotewold and Albin 2024). PD incidence rises with age, peaking in the eighth decade of life, and males generally exhibit higher incidence and prevalence than females. PD is clinically characterized by bradykinesia, resting tremor, stiffness, and postural instability, in addition to various non-motor manifestations that affect quality of life (Mohammed et al. 2024). The persistent degeneration of nigral dopaminergic neurons, which is directly linked to the accumulation of Lewy bodies, is the main pathological characteristic of PD (Desouky et al. 2023). The occurrence of insoluble amyloid fibrils leads to the formation of these Lewy bodies due to the misfolding of α-synuclein (Poewe et al. 2017). This accumulation of protein aggregates gradually leads to neuronal death, a hallmark of neurodegeneration, along with protein imbalance (Desouky et al. 2024; Kurtishi et al. 2019).

In addition to their direct neurotoxicity, α-synuclein aggregates are strong innate immune response activators that stimulate microglial activation and neuroinflammation (Li et al. 2020). Interestingly, these inflammatory responses may not be restricted to the CNS; evidence that peripheral inflammatory responses may contribute to the pathophysiology of PD, with the gastrointestinal system emerging as a considerable contributor to immunological and metabolic disorders associated with the disease (Klann et al. 2022; Pfaffinger et al. 2025).

Growing clinical and mechanistic evidence implicates the gut microbiota in PD pathogenesis via the gut–brain axis (GBA), a bidirectional network of neuronal, immunological, endocrine, and metabolic interactions connecting gut microbiota to the CNS Research indicates that patients with PD have a distinct gut microbiota compared with healthy individuals, characterized by reduced short-chain fatty acid (SCFA)-producing bacteria and increased pro-inflammatory taxa (Klann et al. 2022; Pfaffinger et al. 2025). Dysbiosis is associated with disrupted intestinal barrier integrity, translocation of the endotoxin lipopolysaccharide (LPS) to systemic circulation, and peripheral immune activation that may propagate inflammatory signaling to the CNS via the GBA (George et al. 2025; Nielsen et al. 2021). Recent evidence further supports the role of the gut microbiota influencing neurodegenerative conditions through microbiome–immune–neuronal interactions (Gamal et al. 2026). Microbiome-immune interactions have been shown to affect neuronal and cognitive health in dementia and age-related cognitive decline, whereas disrupted immune balance may lead to neuroinflammation and blood–brain barrier dysfunction (Prajapati et al. 2025a). Moreover, probiotic, prebiotic, postbiotic, and dietary interventions have been proposed to preserve cognitive and neuronal function by regulating gut microbial composition, immune signaling, oxidative balance, and neuroprotective pathways (D’Anniballe De Salles et al. 2026). Consistently, a human-origin probiotic cocktail containing *Lactobacillus* and *Enterococcus* strains decreased Aβ burden, neuroinflammation, microglial activation, BBB disruption, and Alzheimer’s disease-like pathology in APP/PS1 mice (Prajapati et al. 2025b).

The nucleotide-binding oligomerization domain-like receptor protein 3 (NLRP3) inflammasome, which is a cytosolic multiprotein assembly that controls the generation of pro-inflammatory cytokines such as interleukin (IL)-1β and IL-18, triggering cell death through caspase-1, is essential to this connection (Fawzi et al. 2024; Habib et al. 2023; Ibrahim et al. 2021). Preclinical and clinical studies showed the association between the inflammasome signaling pathway and PD (Aldossary et al. 2026; Yang et al. 2026). Moreover, dopaminergic neurodegeneration has been commonly associated with aberrant activation of the inflammasome, which acts as a link between gut dysbiosis, systemic inflammation, and neurological diseases (Zhao et al. 2021), Additionally, through promoting or inhibiting caspase-1 cleavage and affecting the generation of proinflammatory cytokines, the gut microbiome and its metabolites interact with the NLRP3 inflammasome, leading to CNS illness through the gut-brain axis (Yang et al. 2023).

Various metabolic-modulating therapeutics have shown promise in preclinical animal models for the treatment of PD by targeting mitochondrial dysfunction, oxidative stress, and neuroinflammatory signaling. In context, it has been reported that coenzyme Q10 conserved mitochondrial function and boosted atorvastatin activity in a 6-hydroxydopamine-induced dopaminergic toxicity model in rats (Prajapati et al. 2017). In addition, antidiabetic drugs have shown promising effects in PD. Metformin has been shown to reduce dopaminergic neurodegeneration, oxidative stress, neuroinflammation, and mitochondrial dysfunction (Lu et al. 2016; Patil et al. 2014). Additionally, dipeptidyl peptidase-4 inhibitors like vildagliptin have been shown to alleviate behavioral and neurochemical changes linked to dopaminergic neurodegeneration through modulation of RAGE/NF-κB and Nrf2 signaling pathways (Abdelsalam and Safar 2015). These findings indicate that targeting metabolic dysfunction, neuroinflammation, oxidative stress, and mitochondrial impairment could be a promising therapeutic strategy in PD.

Among these antidiabetic and metabolic-targeting therapies, Sodium-glucose cotransporter-2 (SGLT-2) inhibitors are effective in managing hyperglycemia in type 2 diabetic patients (Jasleen et al. 2023). In addition to decreasing body weight and increasing peripheral insulin sensitivity, this class also enhances brain mitochondrial function, lowers neurodegeneration, and modulates the gut microbiota (George et al. 2026; Pawlos et al. 2021). Different SGLT2 inhibitors, including empagliflozin, have displayed favorable neuroprotective effects in preclinical PD models. Canagliflozin (CANA), a member of this family, has also demonstrated promising potential for treating neurological disorders, in addition to its antidiabetic properties. It has been shown to protect against Alzheimer’s disease by suppressing SGLT2 and acetylcholinesterase (Rizvi et al. 2014), while also alleviating neuroinflammation and oxidative stress in an experimental autism model (Nakhal et al. 2022). Moreover, CANA has been shown to restore the intestinal microbiota by increasing beneficial bacterial species such as *Bifidobacterium*,* Lactobacillus*,* Ruminococcus*,* and Blautia*, indicating its ability to attenuate gut microbial imbalance (George et al. 2026; He et al. 2024). Although several of these effects are shared across SGLT2 inhibitors, CANA may exhibit additional influence on gut–microbiota interactions due to its partial inhibition of intestinal SGLT1. In a recent study, it has been shown that CANA increases the availability of glucose in the colon, restores the expression of tight junction proteins, and decreases the accumulation of gut-derived toxins. These effects have been linked to intestinal SGLT1 inhibition (Matsui et al. 2023).

Accordingly, the present study aimed to explore the neuroprotective potential of CANA in a rotenone (ROT)-induced rat model of PD, focusing on whether modulation of gut microbial imbalance and regulation of the interplay between gut microbiota, intestinal barrier function, systemic inflammation, and NLRP3 inflammasome‑related pathways could contribute to its mechanistic effects.

## Materials and methods

### Animals

This animal research was conducted in accordance with the ARRIVE criteria and the ethical principles for the care and use of laboratory animals outlined in the United Kingdom Animals Act (1986) guidelines. The research ethical committee of Ain Shams University’s Faculty of Pharmacy, Egypt, approved the experimental procedure (Approval No. ACUC-FP-ASU RHDIRB2020110301 REC#303). 180–200 gm male Wistar rats have been purchased from the Egyptian Nile Co. for pharmaceutical and chemical industries, Cairo, Egypt. The animals were allowed to acclimate for two weeks before treatment. The animals were housed in an air-conditioned environment with a 12-hour light-dark cycle and had unlimited access to water and food.

### Drugs and chemicals

ROT was obtained from Sigma Aldrich, USA. CANA was obtained as Invokana^®^ from Janssen Pharmaceuticals. Sigma-Aldrich (St. Louis, Mo., USA) provided Methoxyamine HCl, pyridine, MSTFA (N-methyl-N (trimethylsilyl)trifluoroacetamide) with 1% (vol/vol) trimethylchlorosilane, acetonitrile (99.8%), and xylitol [an internal standard for relative quantification using gas chromatography–mass spectrometry (GC/MS)].

### Experimental design

Rats were randomly divided into 3 experimental groups, 15 rats in each group. The first group served as a negative control, receiving subcutaneous sunflower oil and 0.5% CMC in distilled water orally daily for 30 days. The second group received ROT (2 mg/kg subcutaneously, along with 0.5% CMC in distilled water orally, one hour later, daily for 30 days). The third group received ROT (2 mg/kg) subcutaneously daily, and after 1 h, rats received CANA (20 mg/kg) orally daily for 30 days (He et al. 2024; Mohammed et al. 2024).

In line with established ROT-induced PD models, the ROT dosage and duration were chosen to cause chronic PD-like pathology, including oxidative damage, neuroinflammatory activation, mitochondrial failure, nigrostriatal changes, and motor impairment (Johnson and Bobrovskaya 2015). CANA was administered orally at 20 mg/kg based on previous preclinical studies in rats, affecting gut microbiota imbalance and gut-derived toxins, and gut–brain inflammatory signaling (He et al. 2024). The 1-h interval between ROT and CANA administration was used to evaluate CANA effects during ongoing ROT exposure rather than as a post-induction therapeutic protocol.

Afterwards, on day 30, fecal pellets were aseptically collected after each rat was individually placed in a cage presterilized with 70% alcohol, and then pooled fecal samples were taken from each group and instantly preserved at -80 °C until further analysis. Subsequently, to evaluate motor function, behavioral tests including locomotor activity, rotarod tests, and catalepsy tests were performed blindly for rats. Before scarification, blood samples were taken via orbital puncture while rats were under anesthesia with thiopental sodium (40 mg/kg) administered intraperitoneally. After centrifugation for 10 min at 4500 rpm at 4 °C, the separated serum samples were immediately stored at -80 °C for future examination. Then the rats were sacrificed via cervical dislocation (following anesthesia), and their skulls were split on ice. Brains and colon tissues from each group were fixed in formalin (pH = 4) and embedded in paraffin for histological and immunohistochemical assessments. In addition, the midbrains and striata were dissected out on ice. Specimens from all groups were promptly snap-frozen and preserved at −80 °C for upcoming biochemical analysis.

### Body weight

Rat body weights were continuously tracked while each rat received the same amount of chow every day. According to a previous study, the percentage of body weight change was calculated (Fawzi et al. 2024).

### Blood glucose measurement

An Accu-chek glucometer was used to test blood glucose levels on the first day, representing the initial blood glucose, and on the last day, two hours after drug administration. The blood glucose percentage change was subsequently calculated (George et al. 2021).

### Behavioral assessments

To reduce observational bias, behavioral assessments were performed by an investigator who was blinded to the treatment allocation.

#### Locomotor activity

Before locomotor recording, each rat was permitted to habituate with the testing apparatus for 2 min. Utilizing the Opto-Varimex–Mini Model B (Columbus, OH, USA), the animal’s locomotor activity was evaluated. It is made up of 15 infrared beams, which are located 5 cm apart, each with a wavelength of 875 nm and a diameter of 0.32 cm. Rat movements were detected and counted as interruptions to I-R beams and the results were expressed as counts per 5 min (Elhawary et al. 2025).

#### Rotarod test

A rotarod instrument (model 3375-R4, TSE systems) with rotating bars was used to measure the motor coordination of rats. Rats underwent training and assessment sessions; the 3 training sessions were conducted at 4 rpm for 60 s separated by 10 min. Subsequently, the speed of the rotating bars increased from 4 to 40 rpm over two minutes during the assessment session. The latency for each rat to fall was determined with a cutoff time of 120 s. Each rat was evaluated in three separate sessions (Liu et al. 2015).

#### Catalepsy tests (Grid and Bar)

The grid and bar tests were performed on animals 24 h following their last treatment to assess muscular rigidity. In the grid test, rats were positioned with their four paws in the center of a 12 × 12 cm mesh grid with 0.5 × 0.5 cm apertures situated 20 cm above the surface. The time it took for each rat to descend was called descent latency (Zhang et al. 2017). In the bar test, rats were carefully positioned so that they could grasp with their forepaws the bar, which was 10 cm above the ground. Each rat’s latency to remove one or both paws was documented, and for both tests, the cutoff period was 30 s. Three consecutive trials were conducted for each animal, and the average latency was computed for analysis (Neely et al. 2013).

### Histological examination

After being fixed in 10% neutral buffered formalin for 24 h, the brains and colon from each group were washed and subjected to serial dilutions of ethanol to dehydrate them. Furthermore, these samples were washed and embedded in paraffin for 24 h at 56 °C. Blocks of paraffin were cut into slices that were 4 μm in thickness. After being mounted onto glass slides, these sections that represented the different brain regions and others for the colon were deparaffinized. Finally, a hematoxylin and eosin stain was applied to these slides and then visualized by utilizing a full-HD microscopic camera and the Leica application for tissue section analysis (Leica Microsystems GmbH, Wetzlar, Germany). For each animal, 15 non-overlapping sections from similar anatomical levels were evaluated. Neuronal degeneration was determined using recognized histological criteria, which included neuronal shrinkage, cytoplasmic eosinophilia, pyknotic nuclei, and loss of normal neuronal architecture (Bancroft and Gamble 2008).

### Immunohistochemical analysis

Sections of the previous paraffin-embedded tissues (4 μm thick) were deparaffinized, rehydrated, and blocked. The brain sections were incubated overnight at 4 °C with an anti-α-synuclein antibody (sc-12767, Santa Cruz Biotechnology, Inc, Texas, USA). The same procedures were applied to the colonic section with anti-Claudin-1 antibody (MH25 71-7800) purchased from ThermoFisher Scientific, USA. Following the incubation of the primary antibody, the sections underwent counterstaining and sequentially treated with streptavidin-HRP and biotinylated secondary antibodies. Utilizing a Leica DM500 microscope (Wetzlar, Germany), slides were inspected. With three rats per group (*n* = 3), images of the striata, substantia nigra, and colonic regions were taken. The brown immune-positive areas were then measured as optical density (OD) using ImageJ software (version 1.46a; NIH, USA) across multiple high-power fields per section. At the same magnification, 15 non-overlapping microscopic fields from each segment were examined, and quantification was performed by an investigator blinded to treatment allocation (George et al. 2024).

### Microbiome profiling

#### DNA extraction and quantification

To investigate any potential gut microbiota alterations, as well as any potential protective effect exerted by CANA, DNA was extracted from nine samples, representing three pooled biological replicates per group. Each pooled biological replicate involved fecal material carefully collected from four or five rats. The microbial DNA was extracted using the QIAamp Fast DNA Stool Mini Kit according to the manufacturer’s protocol (Qiagen, Hilden, Germany) and immediately stored at -20 °C. A nanodrop spectrophotometer was used to assess the nucleic acid concentration and purity (260/280 and 260/230 ratios) of each sample. Concentrations were further quantified by Qubit^®^ fluorometer (Life Technologies, Carlsbad, CA, USA) using the Qubit dsDNA Hs assay kit (Life Technologies Corporation, Oregon, USA).

#### 16S rRNA amplicon sequencing and analysis

The high-quality, concentrated DNA from each of the nine fecal samples was sequenced at M.A.R.C. research center in Cairo, Egypt, using the MiSeq platform (Illumina, San Diego, USA), following the Illumina 16S rRNA library preparation protocol. The raw sequence reads were primarily analyzed by MOTHUR software package v.1.36.1 (Schloss et al. 2009), based on the MiSeq standard operating procedure (URL: https://mothur.org/wiki/miseq_sop) (Caporaso et al. 2012; Kozich et al. 2013). Short reads, sequences containing unknown nucleotides (Ns), and those with at least one ambiguous base or more than eight homopolymers were excluded. The Wang approach (Wang et al. 2007) was employed for the Mothur taxonomic assignment against SILVA bacterial database, version 138 (Quast et al. 2012). Subsequently, following a 97% cutoff for sequence similarity, the sequences were clustered into operational taxonomic units (OTUs), and UCHIME was used to detect and eliminate chimeric sequences (Edgar et al. 2011). MOTHUR output files (OTUs abundance and consensus taxonomy), were then analyzed by MicrobiomeAnalyst 2.0—an online platform for the statistical and visual analysis of microbiota data (Lu et al. 2023). Reads were initially denoised using filtering criteria that required a minimum prevalence of 20% and a minimum count threshold of four. Before the final differential abundance analysis, sample readings were rarefied and standardized to the minimum library size. Data scaling, also known as total sum scaling, was then performed and the standard deviation low-variance filter was set at 5%. The Kruskal-Wallis test was conducted for significance testing and for measuring the observed OTUs, Chao1, ACE richness estimators, Simpson, and Shannon’s indices, which are important indices for alpha diversity profiling.

The permutational multivariate analysis of variance (PERMANOVA) statistical approach, based on Bray-Curtis dissimilarity, was used to assess beta diversity, which was then visualized using principal coordinate plots. Using default cutoffs, taxa with significant differences were identified using linear discriminant analysis effect size (LEfSe). The false discovery rate (FDR) approach was used to modify *p* values (Segata et al. 2011).

### Metabolites profiling

#### Fecal samples preparation

For metabolomic analysis, fecal samples were carefully collected from five rats for each experimental group. In a sterile tube, about 100 mg of feces were mixed with 900 µL of sterile PBS buffer (pH 7.4). After vortexing, the suspension was centrifuged for ten minutes at 12,000 × g. A 0.45 μm membrane filter was utilized to filter the supernatant.

For GC–MS analysis, 100 µL of fecal filtrate was mixed with 200 µL of ice-cold acetonitrile (100%) and 5 µL of xylitol (1 mg/mL) as an internal standard. Proteins were precipitated by centrifugation at 7000 × g for 15 min. The resulting supernatant was evaporated using a centrifugal vacuum concentrator (Labconco Co., MO, USA) and further dried by freeze-drying. The dried samples were derivatized by adding 50 µL of methoxyamine hydrochloride (20 mg/mL in pyridine) and incubating for 1 h at 60 °C. Subsequently, 100 µL of N-methyl-N-(trimethylsilyl) trifluoroacetamide (MSTFA) containing 1% trimethylchlorosilane (TMS) was added, followed by incubation for an additional 30 min at 60 °C prior to GC–MS analysis.

#### GC–MS analysis

GC–MS analysis was performed using a Shimadzu GCMS-QP 2010 system (Shimadzu Corporation, Kyoto, Japan) equipped with a split/splitless injector and a capillary column (Rtx-5MS, 30 m × 0.25 mm i.d., 0.25 μm film thickness; Restek, USA). The oven temperature was initially held at 60 °C for 1 min, then increased to 325 °C at a rate of 10 °C/min and maintained at 325 °C for an additional 10 min. The injector temperature was set to 280 °C, with helium as the carrier gas at a constant flow rate of 1.30 mL/min. The interface and ion source temperatures were maintained at 280 °C and 250 °C, respectively. Samples (1 µL of 1% v/v dilution) were injected in split mode with a 10:1 split ratio. Mass spectra were recorded in electron ionization (EI) mode at 70 eV, scanning over an m/z range of 40–650.

#### Metabolites identification

Initially, GC/MS peaks were deconvoluted using AMDIS software (https://www.amdis.net). Identification of silylated metabolites was subsequently performed by comparing their retention indices (RIs), calculated relative to an n-alkane series (C8–C40), and by matching their mass spectra against the NIST library database. Peak abundances were quantified using MS-DIAL software following previously established parameters (Gamal et al. 2025). Data processing parameters were set as follows: mass range, 0–1000 Da; MS¹ mass tolerance, 0.5 Da; minimum peak height, 100; sigma value, 0.7; and retention time tolerance for alignment, 0.075 min.

#### Multivariate data analysis

Multivariate data analysis (MVDA) was conducted using both unsupervised methods, including principal component analysis (PCA) and hierarchical cluster analysis (HCA) and a supervised approach, orthogonal partial least squares–discriminant analysis (OPLS-DA), performed with SIMCA 18.0.1 (Umetrics, Umea, Sweden). All variables were Pareto-scaled and mean-centered prior to analysis. PCA, as an unsupervised technique, was applied to obtain an overview of the variance in metabolite profiles across the different groups. In contrast, OPLS-DA was used to validate the PCA findings and to extract detailed information regarding metabolite composition differences among the groups.

The chemometric models were evaluated using R² and Q² parameters, where R² represents the model’s goodness of fit and Q² reflects its predictive capability. Outliers were identified based on the DModX (distance to model), while strong outliers in the OPLS-DA model were detected using Hotelling’s T² test. Model validity was further assessed through a permutation test (200 iterations) to confirm that the class separation observed among the control, PD, and treated groups was not attributable to random variation.

### Assessment of serum biochemical markers

Bio diagnostic, Giza, Egypt company kits were used to albumin (Catalogue No. AB 10 10), and cholesterol (Catalogue No. CH 12 20) levels, in sera from the different rat groups, following the manufacturer’s instructions. Assessment of LPS in sera of different groups was performed using ELISA kit obtained from MyBioSource, San Diego, CA, USA (Catalogue No. MBS261904). Results were presented as ng/ml for LPS and mg/dl for albumin and cholesterol.

### Assessment of oxidative stress and antioxidant defense

To assess redox balance, antioxidant defense markers and oxidative stress indicators were identified colorimetrically in the sera, as well as striatum, midbrain, and colon tissue homogenates using kits obtained from Bio diagnostics, Giza, Egypt. Superoxide dismutase (SOD) enzyme activity was determined by blocking the reduction of nitro-blue tetrazolium dye by phenazine methosulfate for 5 min. The color produced was measured at 560 nm. This technique was consistent with Nishikimi et al., and the results were shown as U/min/gm tissue (Nishikimi et al. 1972). Moreover, the Aebi method was used to evaluate the antioxidant catalase (CAT) activity, and the results are expressed as U/gm tissue (Aebi 1984). Furthermore, the assessment of reduced glutathione (GSH) levels was performed according to the method published by Beutler et al., and results were expressed as mmol/gm tissue (Beutler et al. 1963). For the oxidative stress markers, firstly, in accordance with Aebi, hydrogen peroxide (H_2_O_2_) was assessed in the sample homogenates, and the results were expressed in nM/gm tissue (Aebi 1984). In addition, the degree of lipid peroxidation was assessed by thiobarbituric acid reactive chemicals measured as malondialdehyde (MDA), in compliance with Satoh, and the results were expressed as nmol/gm tissue (Satoh 1978).

Eventually, nitric oxide was evaluated using a kit obtained from Bio diagnostics, Giza, Egypt. In an acidic environment, nitrite reacts to form nitrous acid, which diazotizes sulfanilamide. The resulting compound interacts with N-(1-naphthyl)ethylenediamine, producing a bright reddish-purple azo dye. The intensity of this color, measured at 540 nm, is exactly proportional to the sample’s nitrite concentration, and the results were expressed as µmol/gm tissue.

### Assessment of the inflammasome (NLRP3) pathway

ELISA kits from MyBioSource, USA (Catalogue No. MBS3809440), Elab Science, USA (Catalogue No. E-EL-R0674), MyBioSource, USA (Catalogue No. MBS451267), and abcam, USA (Catalogue No. ab100768), respectively, were used according to manufacturers’ protocols to measure the striatal and midbrain levels of NLRP3, p65 NF-KB, caspase-1, and IL-1β, where NLRP3 and IL-1β levels were expressed as ng/gm tissue, while p65 NF-KB, and caspase-1 concentrations were expressed as pg/gm tissue.

### Statistical analysis

The statistical analysis of data was performed utilizing GraphPad Prism software, version 9.0 (GraphPad Software, La Jolla, CA, USA). The data were analyzed using a one-way analysis of variance (ANOVA) with post hoc comparisons using Tukey-Kramer for body weight and behavioral tests, and the standard Tukey test for the remaining parameters (mentioned in each figure legend). The significance criterion was set at a probability level of 0.05 (the exact *p* values were represented on each graph for each comparison between groups).

## Results

### Effect of CANA treatment on body weight in ROT‑induced PD in rats

**Fig. 1:**
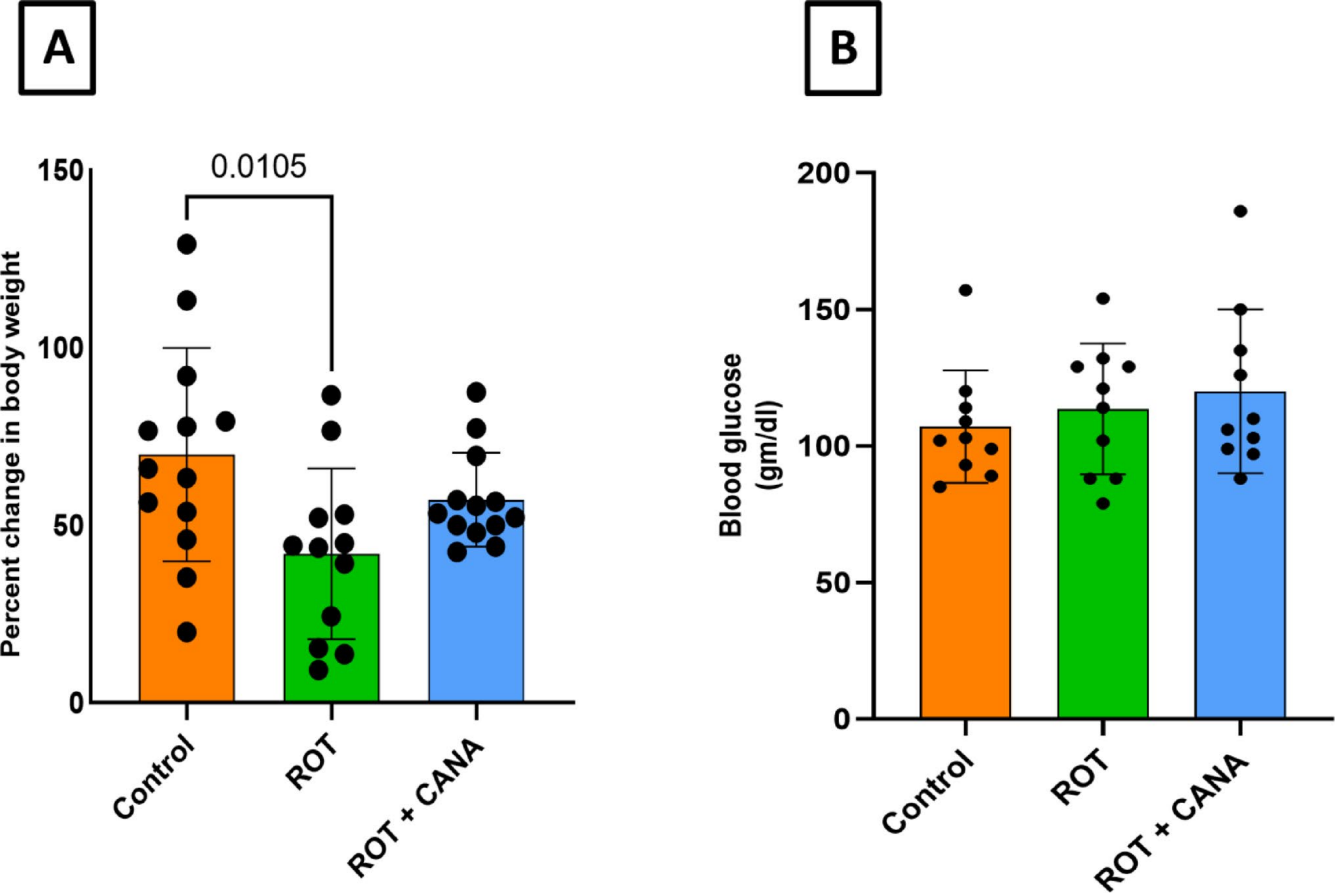
Effect of CANA treatment on body weight (**A**) and blood glucose level (**B**) in rotenone‑induced Parkinson’s disease in rats. Data are presented as mean ± SD (*n* = 10–13). Statistical significance was assessed using one-way analysis of variance (ANOVA) followed by Tukey and Tukey-Kramer as a post-hoc test, respectively. *Rotenone: ROT; Canagliflozin: CANA*

The rats’ body weight change, expressed as a percentage, was employed to reflect on alterations in body weight (Fig. 1A). In comparison with the control group, ROT administration significantly reduced the percentage change in body weight by 1.66-fold. On the other hand, in the CANA-treated group, when compared to the ROT-treated animals, there was an insignificant elevation in percentage body weight.

### Effect of CANA treatment on blood glucose level in ROT‑induced PD in rats

As demonstrated in (Fig. 1B), no statistically significant alterations between the experimental groups were detected, indicating that systemic glucose levels were not significantly affected by ROT exposure and subsequent CANA treatment.

### Effect of CANA treatment on behavioral performance in ROT-induced PD in rats

#### Locomotor activity

**Fig. 2:**
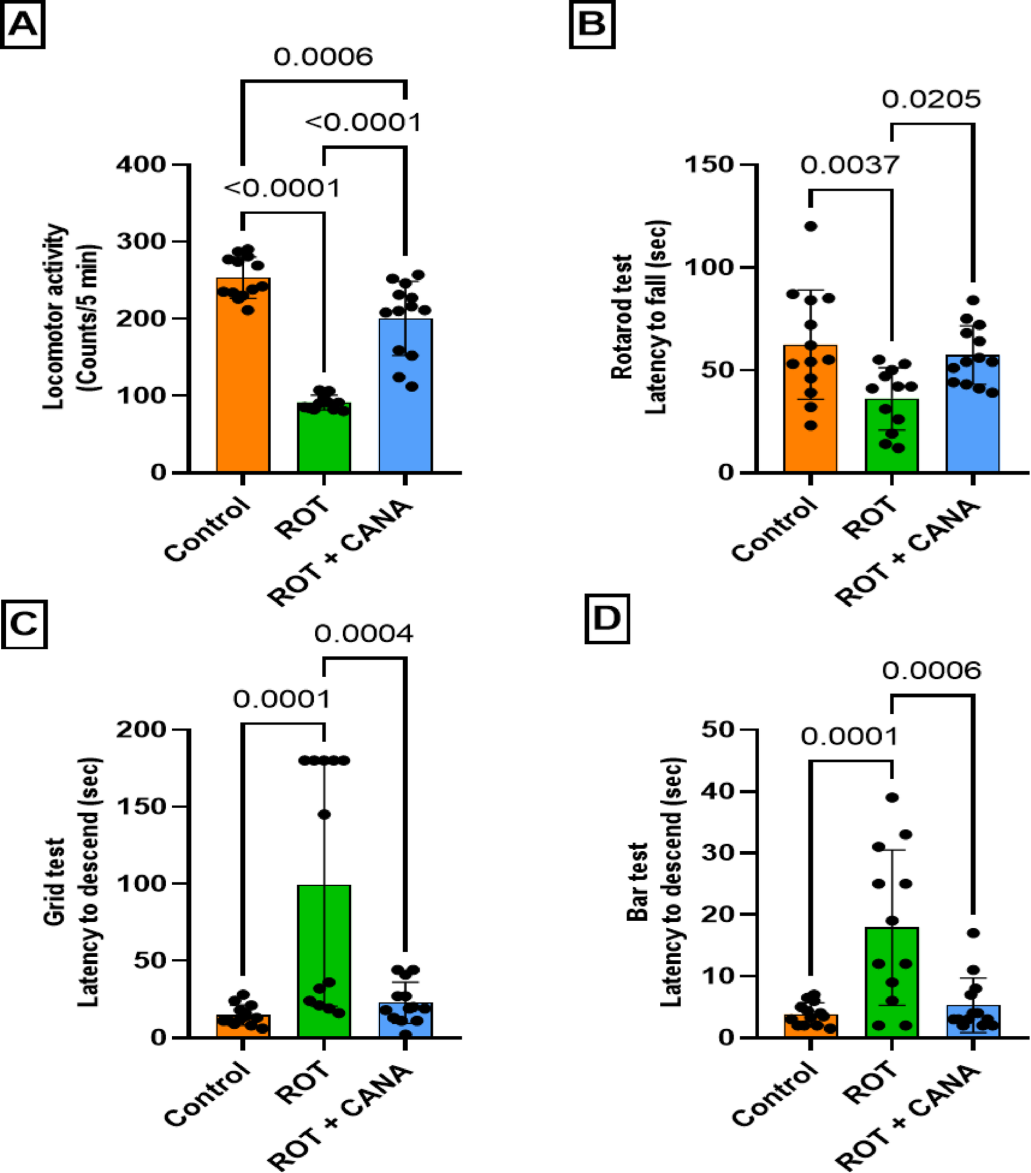
Effect of CANA treatment on Locomotor activity (**A**), rotarod test (**B**), descent latency (Grid test) (**C**), and descent latency (Bar test) (**D**) in rotenone‑induced Parkinson’s disease in rats. Data are presented as mean ± SD (*n* = 12–13). Statistical significance was assessed using one-way analysis of variance (ANOVA) followed by Tukey as a post-hoc test. *Rotenone: ROT; Canagliflozin: CANA.*

Figure 2A depicts a drastic reduction in the locomotor activity by 2.77-fold in ROT-treated rats when compared to the controls. While co-administration of CANA significantly enhanced locomotor activity in comparison to the intoxicated group by 2.19-fold.

#### Motor coordination

A remarkable reduction was demonstrated in the motor coordination of ROT-intoxicated rats, evidenced by a significant 1.74-fold decrease in the latency to fall from the rotarod as compared to the control rats. Contrariwise, CANA treatment augmented motor coordination by 1.59-fold in comparison to PD-induced rats, as shown in (Fig. 2B).

#### Catalepsy assessment

ROT resulted in a 7.1- and 4.64-fold increase in the descent latencies in the grid (Fig. 2C) and bar tests (Fig. 2D) as compared to the vehicle-treated rats. In comparison to the ROT-induced group, co-treatment with CANA significantly shortened the descent latencies in the grid and bar test by 4.51 and 3.37, respectively.

### Effect of CANA treatment on the nigrostriatal histopathology and α-synuclein immunoreactivity in ROT‑induced PD in rats.

**Fig. 3:**
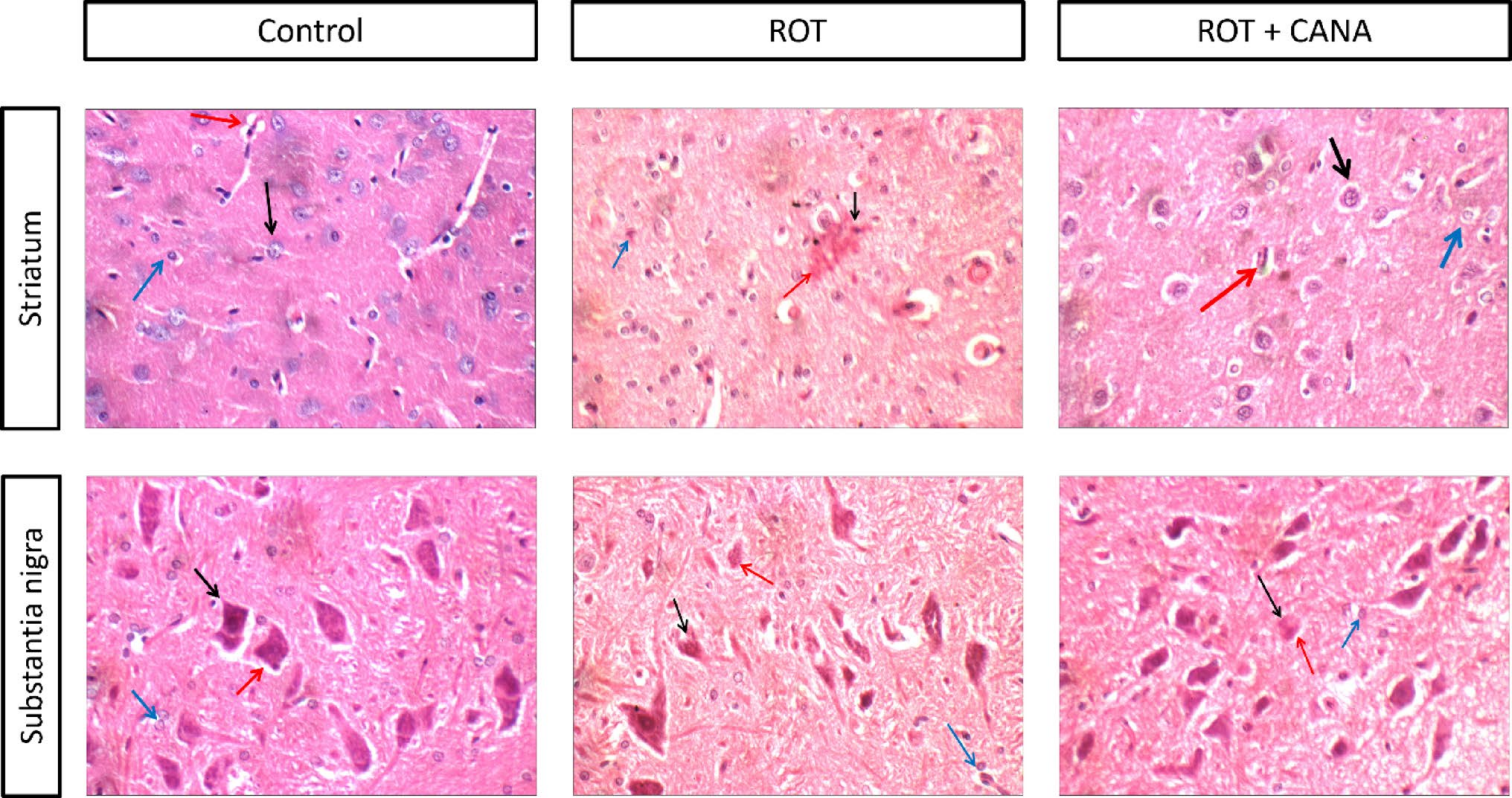
Effect of CANA treatment against rotenone-induced histological alterations of rats in the substantia nigra, and the striatum regions (scale bar 50 μm). Photomicrographs of hematoxylin and eosin-stained sections from the control group (**A**); rotenone-treated group (2 mg/kg) (**B**); rotenone and CANA group (20 mg/kg) (**C**), with 100× magnification power. *Rotenone: ROT; Canagliflozin: CANA.*

Histopathological examination of the SN and striata of the control group displayed the typical histological structure, as shown in Fig. 3. On the contrary, the ROT-intoxicated group showed neuropathological alterations. In the SN, neurodegeneration was prominent, with irregular and fractured neuron cell membranes, nuclear loss in some cells, and eosinophilic cytoplasm with an intra-cytoplasmic inclusion-like structure. The striatum showed markedly degraded neurons, noticeably degenerated glial cells, and eosinophilic plaque-like areas. CANA treatment was found to mitigate these histological alterations compared to the diseased group, in both the SN and striatum. Just a few dispersed neurons showed signs of moderate neurodegeneration, while most neurons retained their normal morphology and distinct nuclei. Glial and vascular structures appeared largely preserved.

**Fig. 4:**
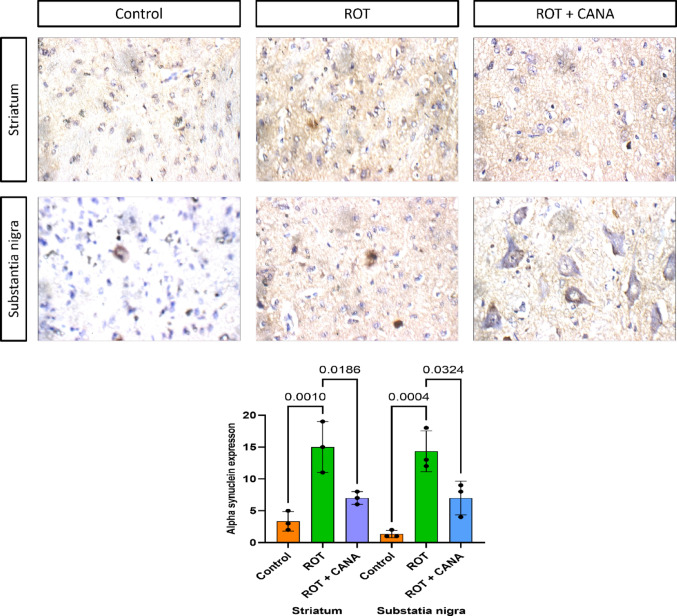
Effect of CANA treatment on the midbrain and the striatum expression of α-synuclein in rotenone‑induced Parkinson’s disease in rats by immunohistochemical staining (100×). Photomicrographs of histological sections from the control group (**A**), rotenone-treated group (2 mg/kg) (**B**), and rotenone and CANA group (20 mg/kg) (**C**). Data are presented as mean ± SD (*n* = 3). Statistical significance was assessed using one-way analysis of variance (ANOVA) followed by Tukey as a post-hoc test. *Rotenone: ROT; Canagliflozin: CANA*

As illustrated in Fig. 4, immunohistochemical analysis further demonstrated that ROT intoxication significantly boosted the striatal expression of α-synuclein by 4.5-fold in comparison to that of the vehicle treatment. Upon CANA cotreatment, the striatal α-synuclein expression was significantly reduced by 1.88-fold relative to ROT treatment. Concerning the SN, the α-synuclein expression level was elevated in rotenone-treated rats relative to the control group. On the other side, CANA treatment showed a significant reduction in the α-synuclein expression compared to the ROT-treated group.

### Effect of CANA treatment on colonic histopathology and claudin-1 expression in ROT‑induced PD in rats

**Fig. 5:**
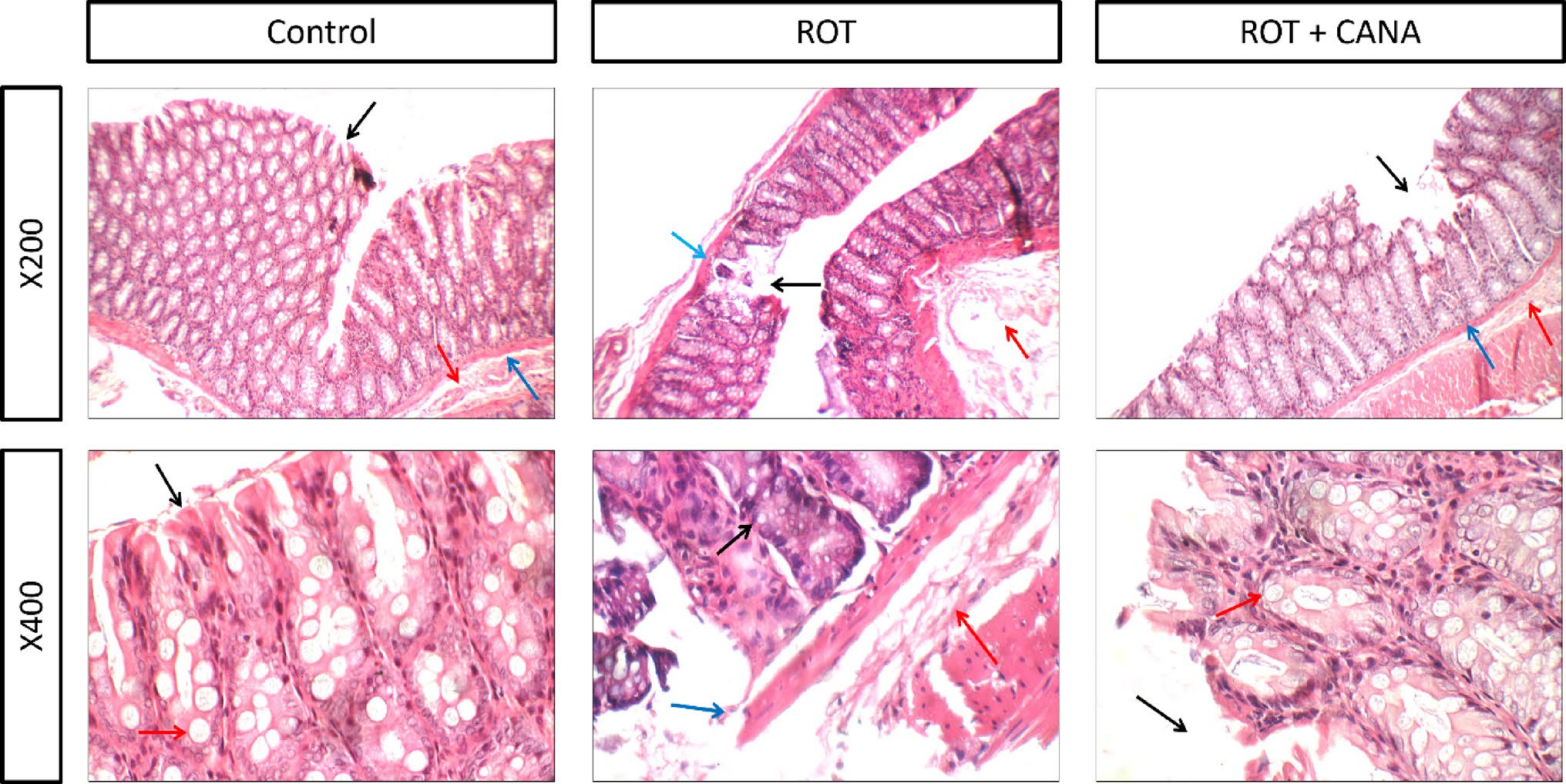
Effect of CANA treatment against rotenone-induced histological alterations of the colon tissue in rats (scale bar 50 μm). Photomicrographs of hematoxylin and eosin-stained sections from the control group (**A**); rotenone-treated group (2 mg/kg) (**B**); rotenone and CANA group (20 mg/kg) (**C**); with ×200 and ×400 magnification power. *Rotenone: ROT; Canagliflozin: CANA*

Colonic tissues were then examined (Fig. 5) to determine if any associated gastrointestinal alterations occurred. The control group displayed normal histological structure characterized by intact viable mucosa with intact superficial layer, and normal goblet cells, muscularis mucosa, and submucosa. Inversely, the colonic tissues of the ROT-treated group revealed ulcerated mucosa with marked reduction of goblet cells, distorted muscularis mucosa with sites of perforation, and markedly edematous submucosa with mild inflammatory infiltrate. Interestingly, CANA co-administration demonstrated substantial preservation of colonic structure, showing mucin-secreting glands containing a normal amount of goblet cells, and intact muscularis mucosa and submucosa.

**Fig. 6:**
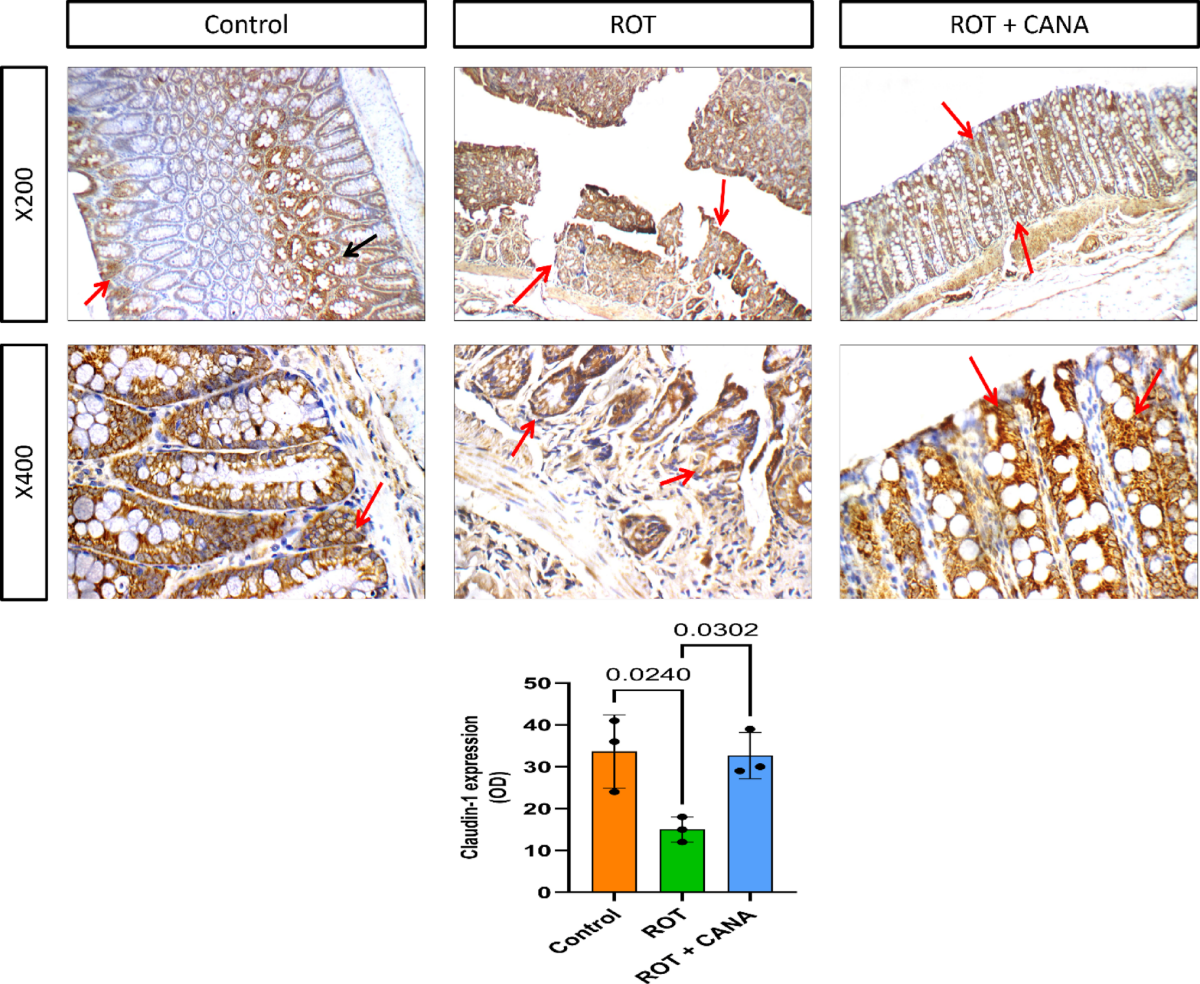
Expression of the colorectal tight junction proteins (claudin-1) by immunohistochemical staining (100× and 40×). Photomicrographs of histological sections from the control group (**A**), rotenone-treated group (2 mg/kg) (**B**), and rotenone and CANA group (20 mg/kg) (**C**). Claudin-1 expression presented as OD. Data are presented as mean ± SD (*n* = 3). Statistical significance was assessed using one-way analysis of variance (ANOVA) followed by Tukey as a post-hoc test. *Rotenone: ROT; Canagliflozin: CANA*

Consistent with impaired intestinal barrier integrity, immunohistochemical analysis was employed to evaluate the intestinal integrity by assessing the expression of the tight junction protein claudin-1 (Fig. 6). In ROT-intoxicated rats, the expression of claudin-1 was remarkably suppressed in comparison to control rats by 2.24-fold. On the contrary, CANA-cotreated rats displayed restored intestinal integrity with significant enhancement in the expression of claudin-1 by 2.13-fold compared to the intoxicated group.

### Effect of CANA treatment on fecal microbiome profiling and microbial diversity of ROT‑induced PD rats

MOTHUR analysis identified 449 taxonomic units of different taxonomic levels (14 phyla, 23 classes, 54 orders, 99 families, and 257 genera) that originated from 532,113 filtered and classified reads with an average of 59,123 reads per sample. All microbiome data were then re-filtered, re-analyzed, and visualized by the MicrobiomeAnalyst 2.0 platform to compare the identified taxa among sample groups at different taxonomic levels, as follows. The analyzed reads were normalized- across all samples- to the total number of reads and then rarefied to the minimum library size (18,141). Subsequently, applying a low-variance filter has removed 6406 low-abundance features and 108 low-variance features. At the phylum level, MOTHUR initially identified 14 phyla, 10 of which were retained by MicrobiomeAnalyst. Three phyla were the most predominant in all samples: *Bacteroidota* (with a mean relative abundance of 88.67% of filtered classified reads), followed by *Firmicutes* (with a mean relative abundance = 8.67% of filtered classified reads), and *Campylobacterota* (with a mean relative abundance = 1.15 of filtered classified reads). Seven other phyla were found in low proportions (Supplementary Fig. 1). At the genus level, 97 genera were observed, 10 of which were the predominant ones (Supplementary Fig. 2), while 87 other genera were found in low proportions.

**Fig. 7:**
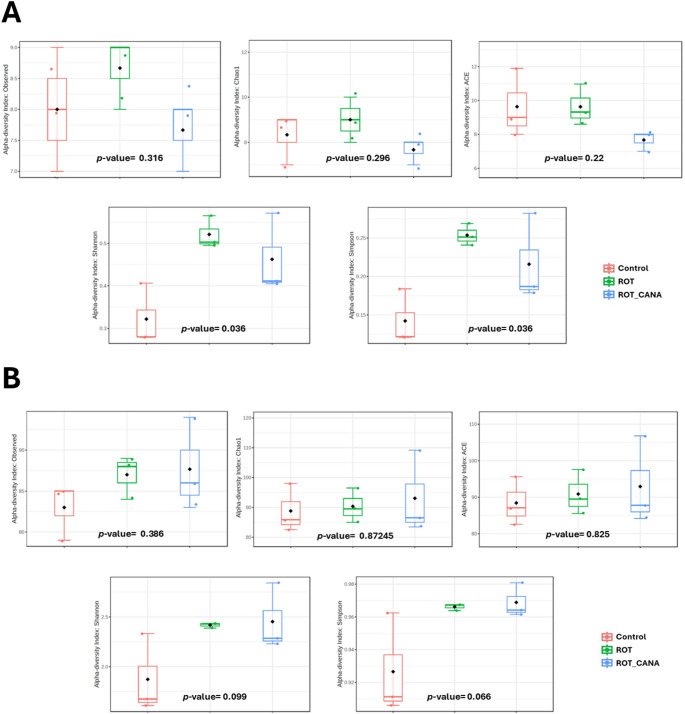
Different alpha diversity metrics (Observed, Chao1, ACE, Shannon, and Simpson diversity indices), for microbiome samples, for different groups, at the phylum level (**A**) and genus level (**B**)

**Fig. 8:**
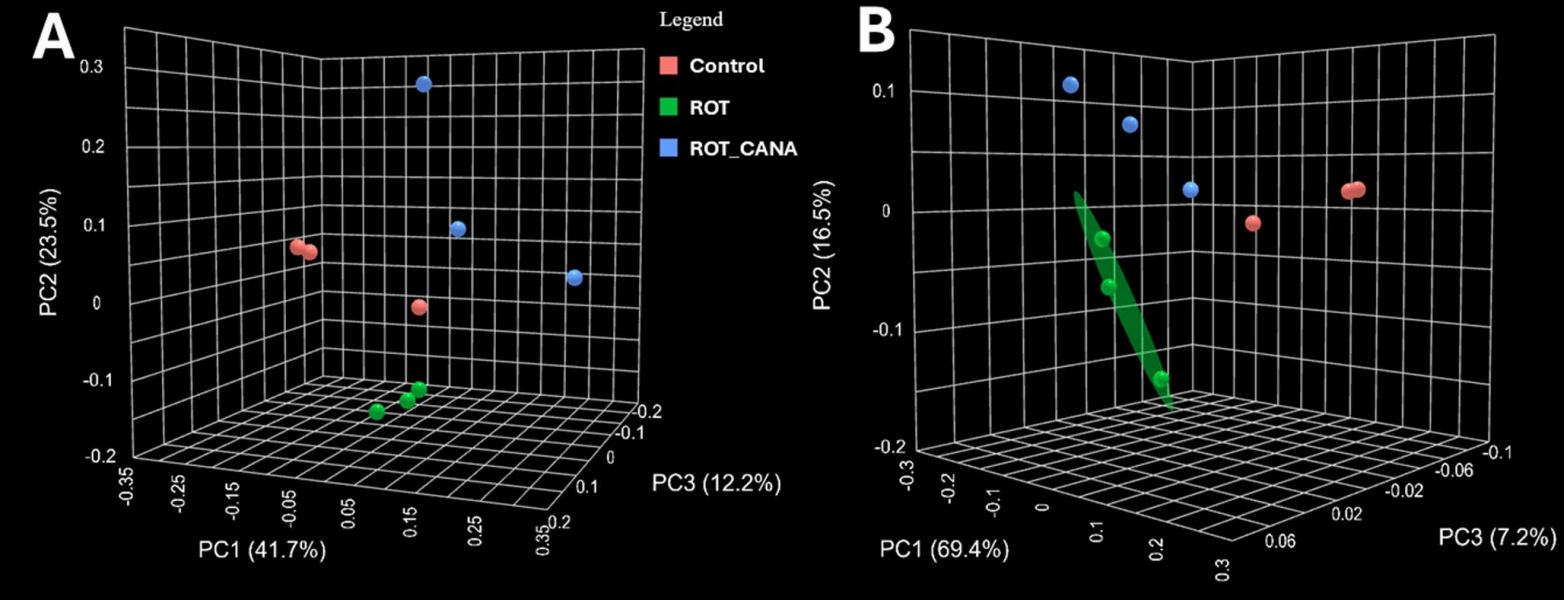
Beta-diversity, visualized by PCA of Bray–Curtis distances between different samples. **A** Comparison between Control, ROT, and ROT+CANA groups at the phylum level: samples are separated into three clusters—PERMANOVA F-value = 3.464, R-squared = 0.58, *p*-value = 0.009. **B** Comparison between Control, ROT, and ROT+CANA groups at the genus level: the samples are separated into three clusters, with the ROT model demonstrating the most distinct one—PERMANOVA F-value = 4.4, R-squared= 0.594, *p* value = 0.009. *Rotenone: ROT; Canagliflozin: CANA*

The alpha diversity of the rat fecal microbiota was estimated by different indices at the phylum and genus levels. While no significant differences were observed between the three groups at the genus level across five alpha diversity indices (Fig. 7B), two indices—Shannon and Simpson—showed significant differences at the phylum level (Fig. 7A), with the control group exhibiting the lowest diversity. The gut microbiota beta diversity, evaluated through principal component analysis (PCA) of Bray–Curtis distances of all sequence variants, revealed clear segregation between different groups at both the phylum and genus levels (Fig. 8A, B, respectively).

**Fig. 9:**
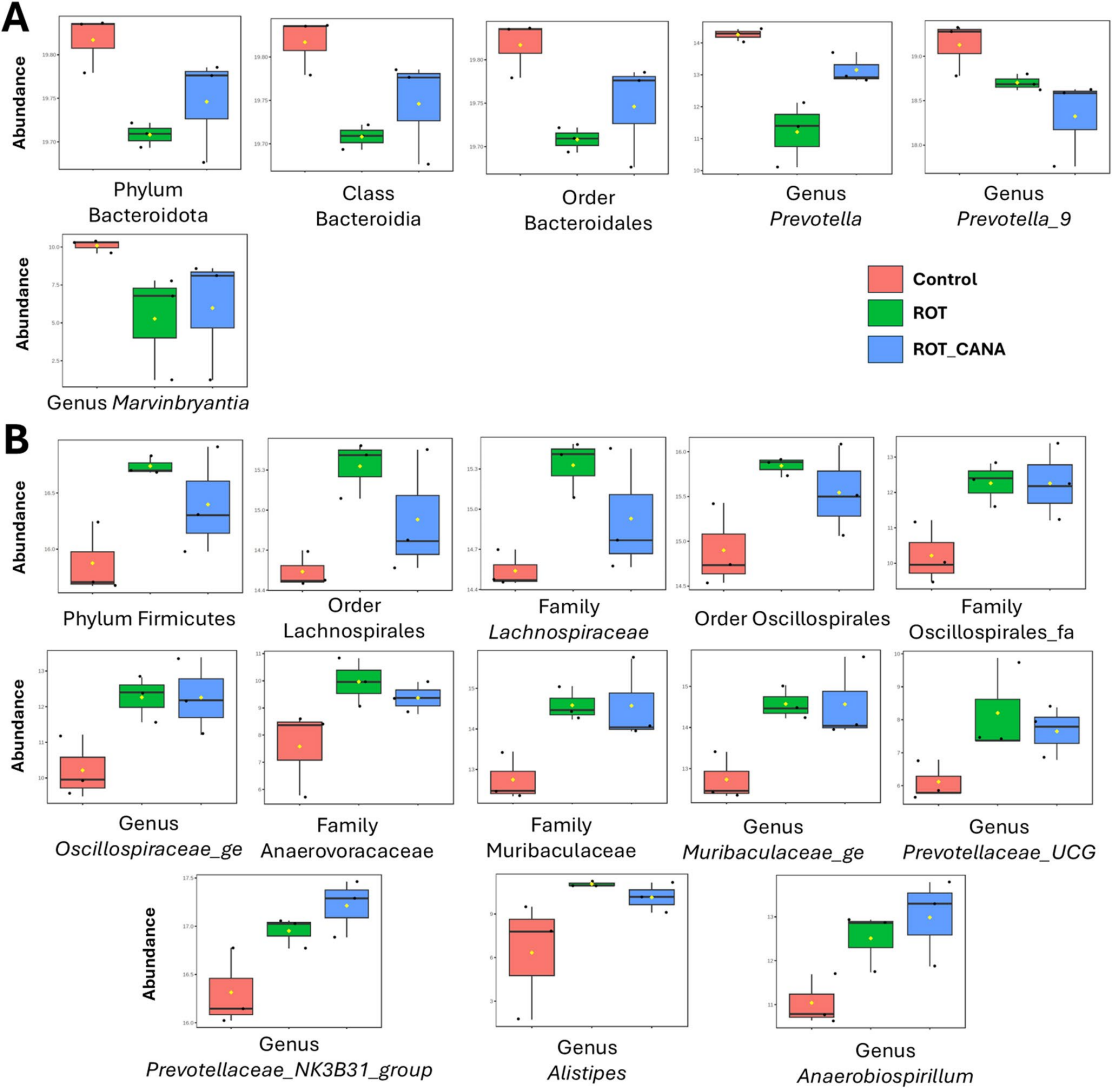
Differential relative abundance of taxa among the three tested groups. The box plots describe the differential relative abundance of nineteen bacterial taxa showing significant differences across groups: abundant taxa in the control group (**A**), and scarce taxa in the control group (**B**). The significance was identified by LEfSe analysis, with *p* value cutoff of 0.05, and *p*-value adjustment by the FDR method. The Y-axis represents relative abundance expressed as log-transformed counts. *Rotenone: ROT; Canagliflozin: CANA*

**Fig. 10:**
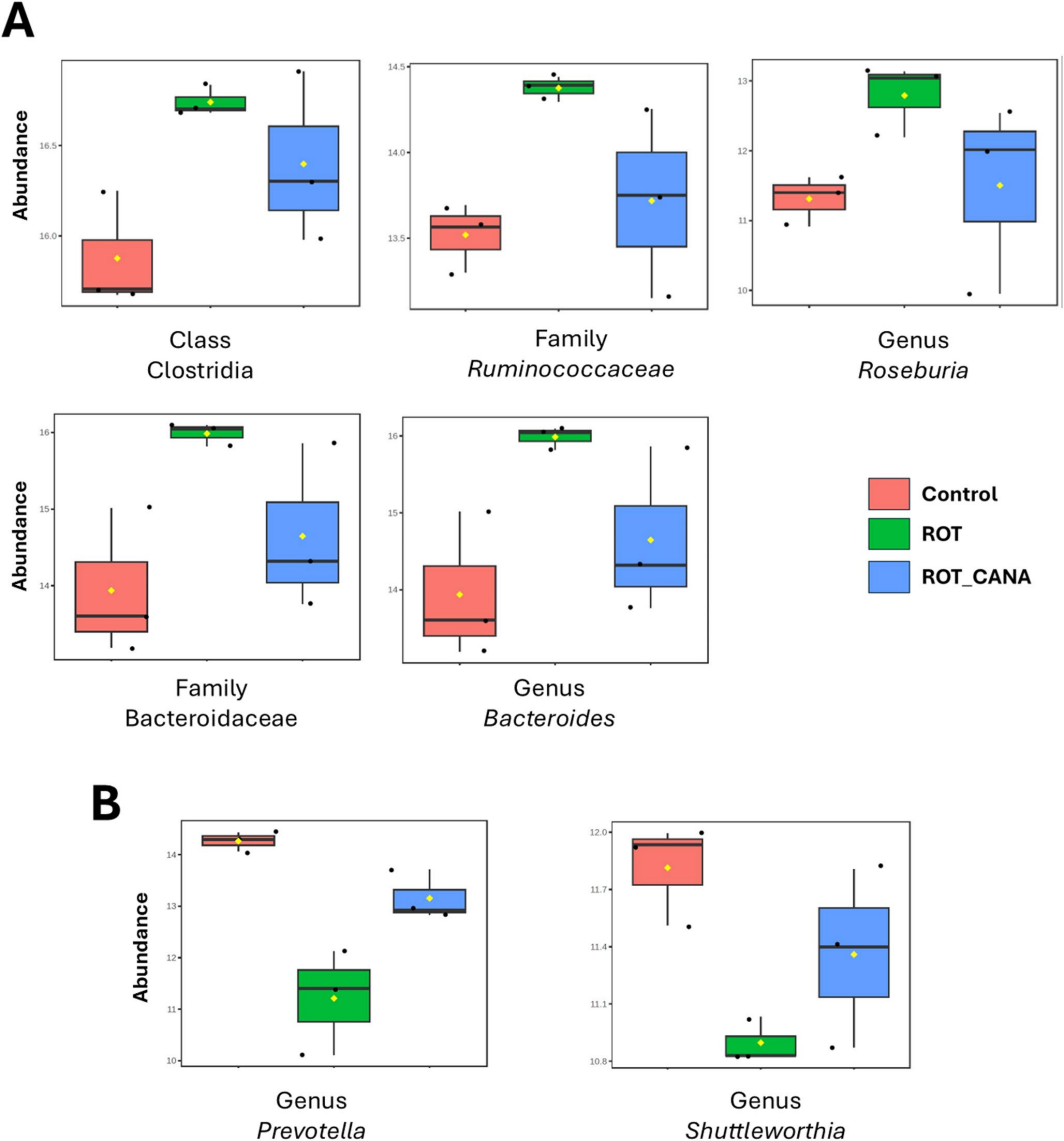
Differential relative abundance of taxa among the three tested groups. The box plots describe the differential relative abundance of seven significant taxa in different samples: abundant taxa in the ROT model group (**A**), and scarce taxa in the model group (**B**). The significance was identified by LEfSe analysis, with a *p*-value cutoff of 0.05, and *p*-value adjustment by the FDR method. The Y-axis represents relative abundance expressed as log transformed counts. *Rotenone: ROT; Canagliflozin: CANA*

A comparative analysis of microbiome profiles across the three groups revealed statistically significant differences in 32 taxa across various taxonomic levels. These differences were identified using the Linear discriminant analysis effect size (LEfSe) method, as illustrated in Figs. 9 and 10. Among these significantly different taxa, phylum *Bacteroidota* (including class *Bacteroidia*, order *Bacteroidales*, genera *Prevotella* and *Prevotella*_9), and genus *Marvinbryantia* were more abundant in the control rats (Fig. 9A). Phylum *Firmicutes* (order *Lachnospirales*, family *Lachnospiraceae*, genera *Lachnospiraceae*_ge and *Lachnospiraceae*_*UCG*_*001;* order *Oscillospirales*, family *Oscillospirales*_*fa*, genus *Oscillospiraceae*_ge; Family *Anaerovoracaceae*), family *Muribaculaceae*, and its genus Muribaculaceae_ge, and genera *Prevotellaceae*_UCG, *Prevotellaceae*_NK3B31_group, *Alistipes* (all belonging to order *Bacteroidales*), and genus *Anaerobiospirillum* were less abundant in the same group (Fig. 9B).

Class *Clostridia* (including family *Ruminococcaceae*, and genus *Roseburia*), and family *Bacteroidaceae* (including its genus *Bacteroides*) were more abundant in the ROT model group (Fig. 10A). Interestingly, genus *Shuttleworthia* -belonging to class Clostridia- and genus *Prevotella*- belonging to class *Bacteroidia*, order *Bacteroidales*-were less abundant in the same group (Fig. 10B). *Prevotella_9* was less abundant in the ROT+CANA group Fig. 9A, while *Anaerobiospirillum* and *Prevotellaceae*_*NK3B31*_group genera were enriched in the same group (Fig. 9B).

**Fig. 11:**
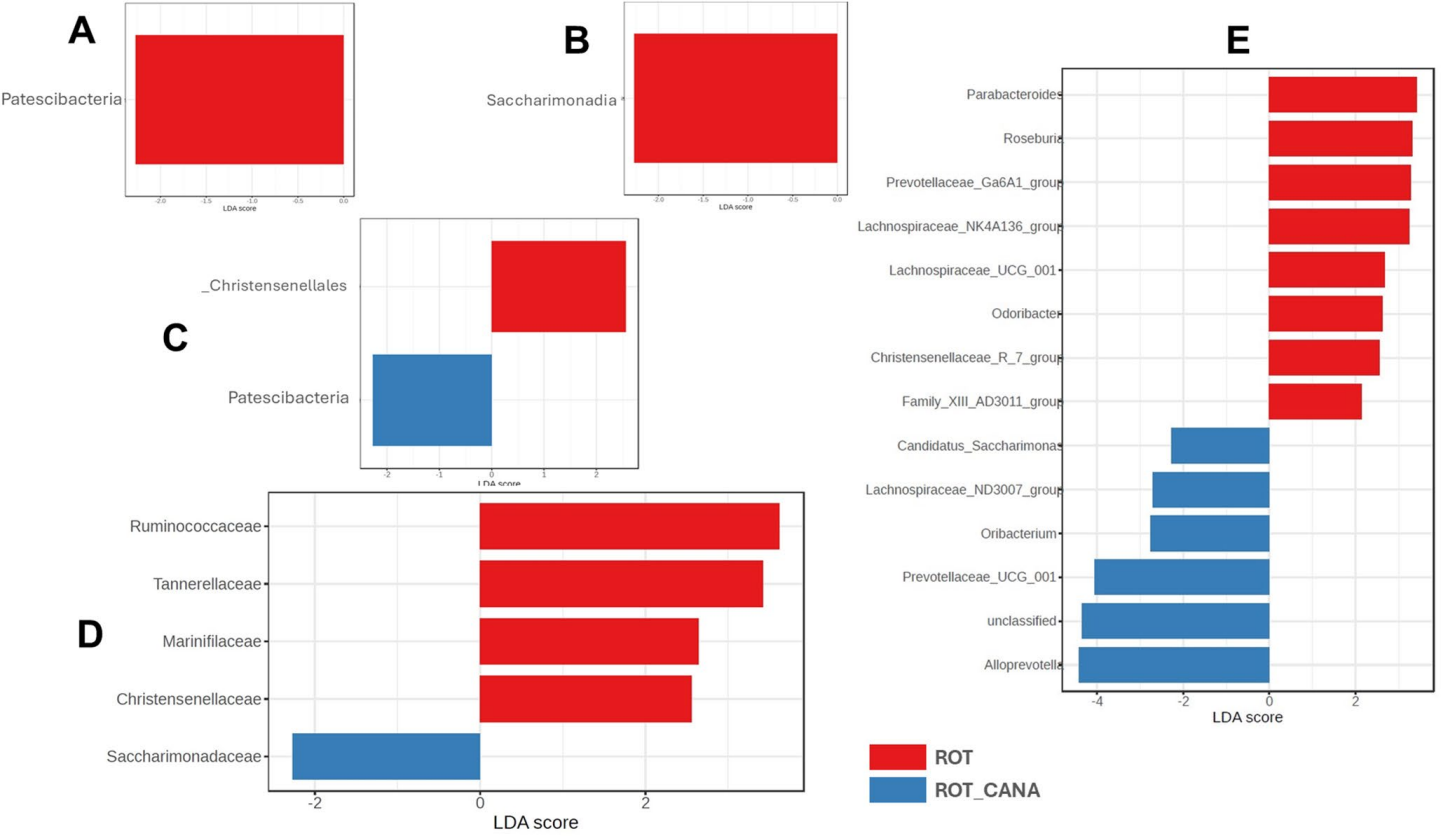
Taxa with significant differences in relative abundance between the two tested sample groups; the length of the bars represents the logarithm of linear discriminant analysis (LDA). Red bars represent enriched taxa in the ROT model group; blue bars represent enriched taxa in the ROT+CANA group. Enriched taxa belong to the phylum level (**A**), class level (**B**), order level (**C**), family level (**D**), and genus level (**E**). *Rotenone: ROT; Canagliflozin: CANA*

Moreover, we compared the fecal microbiome taxonomic composition and diversity of the model group (ROT) with those of the model + treatment group (ROT+CANA) to investigate canagliflozin’s potential microbiome-modulating and therapeutic effects, as well as its ability to ameliorate or reverse PD-driven microbiome alterations. We used LEfSe analysis to determine the differences in the relative abundance of microbial taxa between the ROT and ROT+CANA groups, and the comparative LEfSe analysis revealed statistically significant differences in several taxa across various taxonomic levels, from phylum to genus levels, summarized in Fig. 11.

**Fig. 12:**
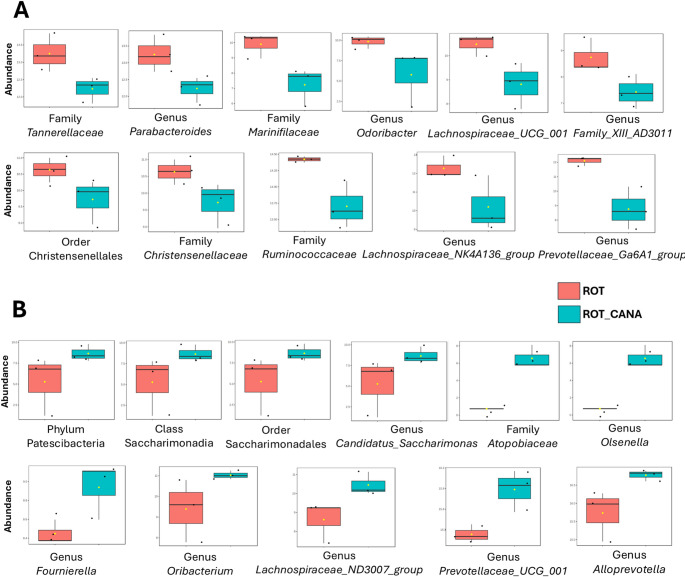
Differential relative abundance of taxa among the three tested groups. Boxplots describe the differential relative abundance of twenty-two significant taxa; abundant taxa in the ROT model group (**A**), and scarce taxa in the same model group (**B**). The significance was identified by LEfSe analysis, with a *p*-value cutoff of 0.05, and *p*-value adjustment by the FDR method. The Y-axis represents relative abundance expressed as log transformed counts. *Rotenone: ROT; Canagliflozin: CANA*

*Parabacteroides* (and its family *Tannerellaceae*), *Odoribacter* (and its family *Marinifilaceae*), which belongs to class *Bacteroidia*, genera Family_*XIII_AD3011*_group, *Lachnospiraceae_NK4A136*_group, *Lachnospiraceae_UCG_001*, and *Prevotellaceae_Ga6A1*_group, and families *Christensenellaceae* (and its order *Christensenellales*), and *Ruminococcaceae*, which all belong to class *Clostridia*, were enriched in the model group. On the other hand, phylum *Patescibacteria* (including class *Saccharimonadia*, order *Saccharimonadales*, and genus *Candidatus*_*Saccharimonas*), and genera *Olsenella* (and its family *Atopobiaceae*), *Fournierella*,* Oribacterium*,* Lachnospiraceae_ND3007*_group, *Prevotellaceae_UCG_001*, and *Alloprevotella* were less abundant in the ROT-treated group but enriched in the CON + ROT group (Fig. 12).

### Effect of CANA treatment on metabolite profiling in ROT‑induced PD in rats

**Fig. 13:**
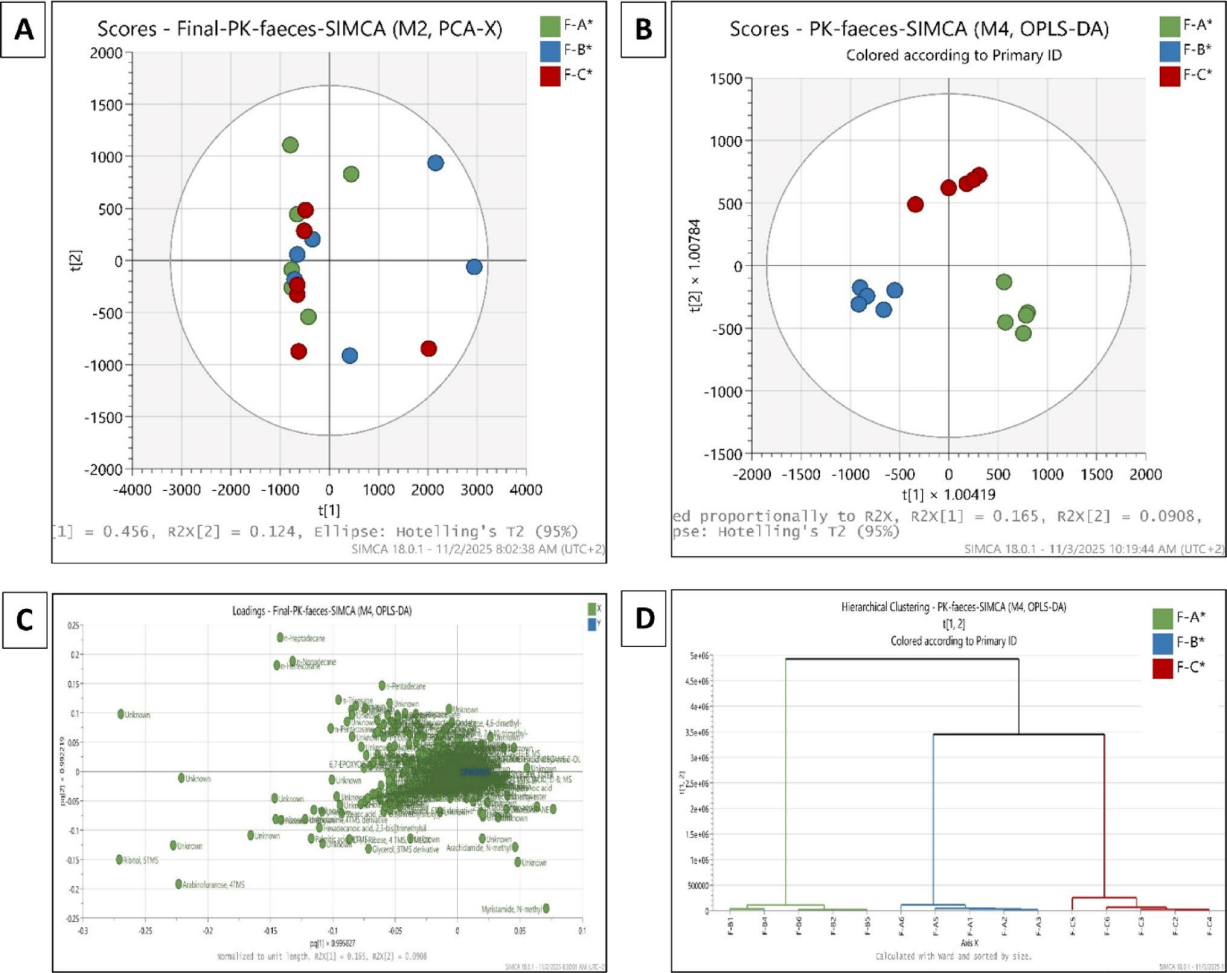
Effect of CANA treatment on metabolite profiling in ROT‑induced PD in rats, GC-MS-based Principal Component Analysis (PCA) score plot (**A**), Orthogonal Partial Least-Squares-Discriminant Analysis (OPLS-DA) scoreplot (**B**), OPLS loading plot (**C**), Hierarchical Cluster Analysis (HCA) (**D**), derived from modeling healthy control rats (F-A), ROT-intoxicated group (F-B), and CANA-treated group (F-C). *Rotenone: ROT; Canagliflozin: CANA*

Unsupervised principal component analysis (PCA) was initially employed to compare overall metabolic differences between the control (A), PD (B), and CANA-treated (C) groups. The first two principal components represented 45.6% and 12.4% of the variation, respectively. As shown in Fig. 13A, PCA didn’t show a clear separation among the investigated groups, with mild metabolic differences insufficient for meaningful differentiation, suggesting that the overall metabolic variations were subtle and partially overlapping. Consequently, OPLS-DA was applied as a more discriminative supervised approach to enhance group separation and identify metabolites contributing most to the observed differences.

The OPLS-DA score plot (Fig. 13B) revealed distinct clustering patterns among the three groups, with the control group positioned in the lower right quadrant, the PD group in the lower left quadrant, reflecting pronounced metabolic deviations associated with disease pathology. and the CANA-treated group appeared in the upper region of the plot, ascending along PC2, suggesting partial metabolic recovery or a shift toward the metabolic state of the control group following treatment.

The OPLS loading plot (Fig. 13C) identified the metabolites contributing most to group separation. Variables located farthest from the origin exerted the strongest influence on discrimination, highlighting key biochemical changes associated with disease pathology and treatment response.

Hierarchical cluster analysis (HCA) further supported these findings, showing a clear separation of the disease group from both the control and treated groups (Fig. 13D). The distinct clustering pattern observed in the HCA dendrogram further confirmed the metabolic differences associated with the disease state. Besides, the proximity of the treated group to the control cluster suggests that treatment induced a partial normalization of the fecal metabolome, indicating that the therapeutic intervention successfully shifted the global fecal metabolome back toward a state of health.

Model parameters indicated significant reliability and predictive performance. The model depicted 77.9% of the variance in the metabolite data (R²X = 0.779) and explained 94% of the variance in group classification (R²Y = 0.94), with a moderate to good cross-validated Q² value of 0.559. Permutation testing (200 iterations) confirmed model validity with an R² intercept of 0.843 and a Q² intercept of −0.6, indicating that class separation was not random chance. Collectively, these results demonstrate that the supervised multivariate analyses effectively distinguished the metabolic profiles of control, disease, and treated groups, providing insight into PD-associated metabolic alterations and CANA treatment effects.

**Fig. 14:**
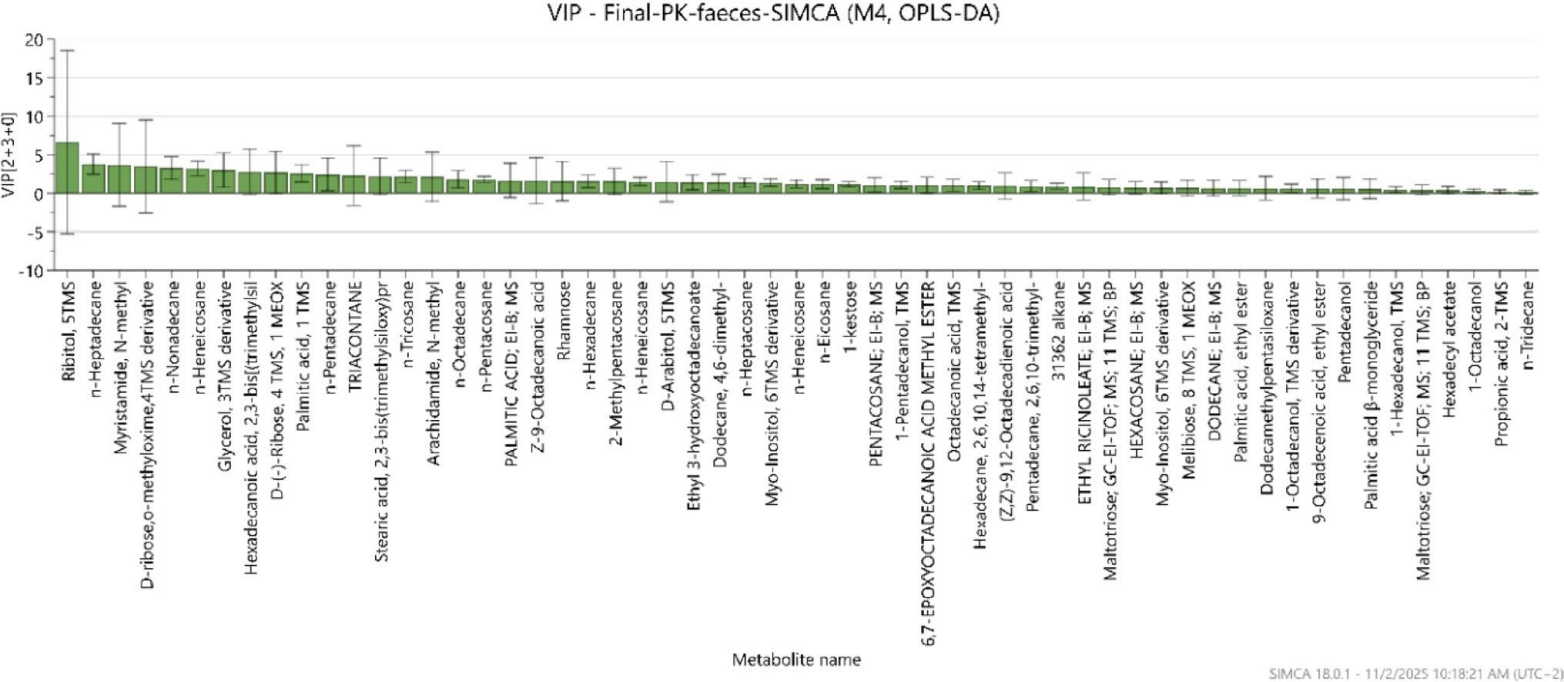
Variable Importance in Projection (VIP) plot identifying keydiscriminatory fecal metabolites

**Fig. 15:**
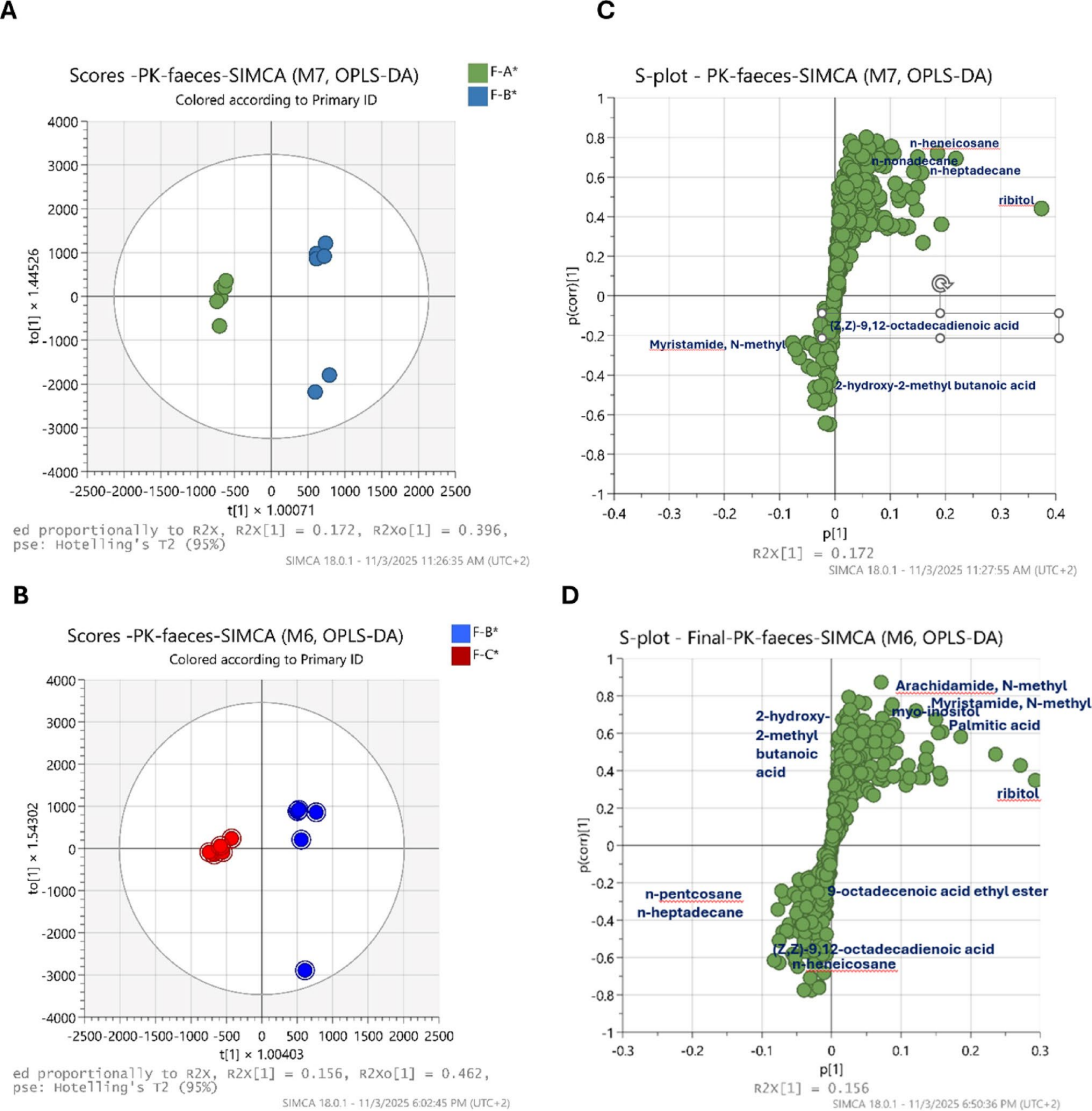
GC-MS-based OPLS-DA score plot **A** derived from modeling healthy control rats (F-A) versus PD group (F-B); **B** derived from modeling CANA-treated group (F-C) versus PD group (F-B); **C**, **D** The respective loading S-plots showing the covariance p [1] against the correlation p (cor)[1] of the variables of the discriminating component of the OPLS-DA mode

To further elucidate the specific biochemical changes underlying these group differences, metabolites with the highest contributions to group separation were identified using VIP plot (Fig. 14). VIP (Variable Importance for the Projection) analysis of the three-group OPLS-DA model identified metabolites with VIP scores > 1 as the most influential contributors to group separation. Among all detected metabolites, ribitol showed the highest VIP score (exceeding 5.0), indicating that it was one of the strongest markers associated with disease progression and treatment response. This was followed by n-alkanes (n-heptadecane, n-nonadecane, n-heneicosane, and n-octadecane) and myristamide, N-methyl (ranked third, with a score approaching 4.0). The VIP analysis also highlighted the broader metabolic shift occurring within the microbiome, conferring high significance scores to other lipid precursors (palmitic acid, arachidamide, N-methyl) as well as specific sugars and sugar alcohols (D-ribose, rhamnose, myo-inositol).To identify metabolites driving group separation, S-plots were generated from pairwise OPLS-DA models (Fig. 15). The resulting S-plots were utilized to visualize the covariance (p[1]) and correlation (p(corr)[1]) of the variables, effectively highlighting the most significant biomarkers driving the separation between groups.

In the comparison between healthy control rats (F-A) and the Parkinson’s Disease (PD) model group (F-B) (Fig. 15A), the S-plot revealed a distinct metabolic shift. The extreme top-right quadrant, representing metabolites significantly elevated in the PD group, was dominated by ribitol. Conversely, the bottom-left quadrant represents the metabolic signature of the healthy state, which was uniquely defined by significant enrichments of 2-hydroxy-2-methylbutanoic acid, myristamide, N-methyl, and (Z, Z)-9,12-octadecadienoic acid (linoleic acid) (Fig. 15C).

Following therapeutic intervention, the OPLS-DA model comparing the CANA-treated group (F-C) to the PD group (F-B) (Fig. 15B) demonstrated a robust metabolic separation, indicating a significant therapeutic shift away from the pathological baseline. To pinpoint the specific metabolites driving this recovery, an S-plot was analyzed (Fig. 15D). The S-plot revealed that the CANA-treated group was characterized by a profound enrichment of fatty acids and long-chain alkanes, specifically (*Z*,*Z*)-9,12-octadecadienoic acid, n-heptadecane, and n-heneicosane. Conversely, the profile of the untreated PD group was dominated by the dysbiosis marker ribitol, alongside a relative elevation of myristamide, N-methyl and 2-hydroxy-2-methylbutanoic acid compared to the treated state. These findings were consistent with VIP scores and loading plot analyses, confirming that the identified metabolites are central to disease pathology and responsive to treatment.

### Effect of CANA treatment on serum LPS, albumin, and cholesterol levels in ROT-treated rats

**Fig. 16:**
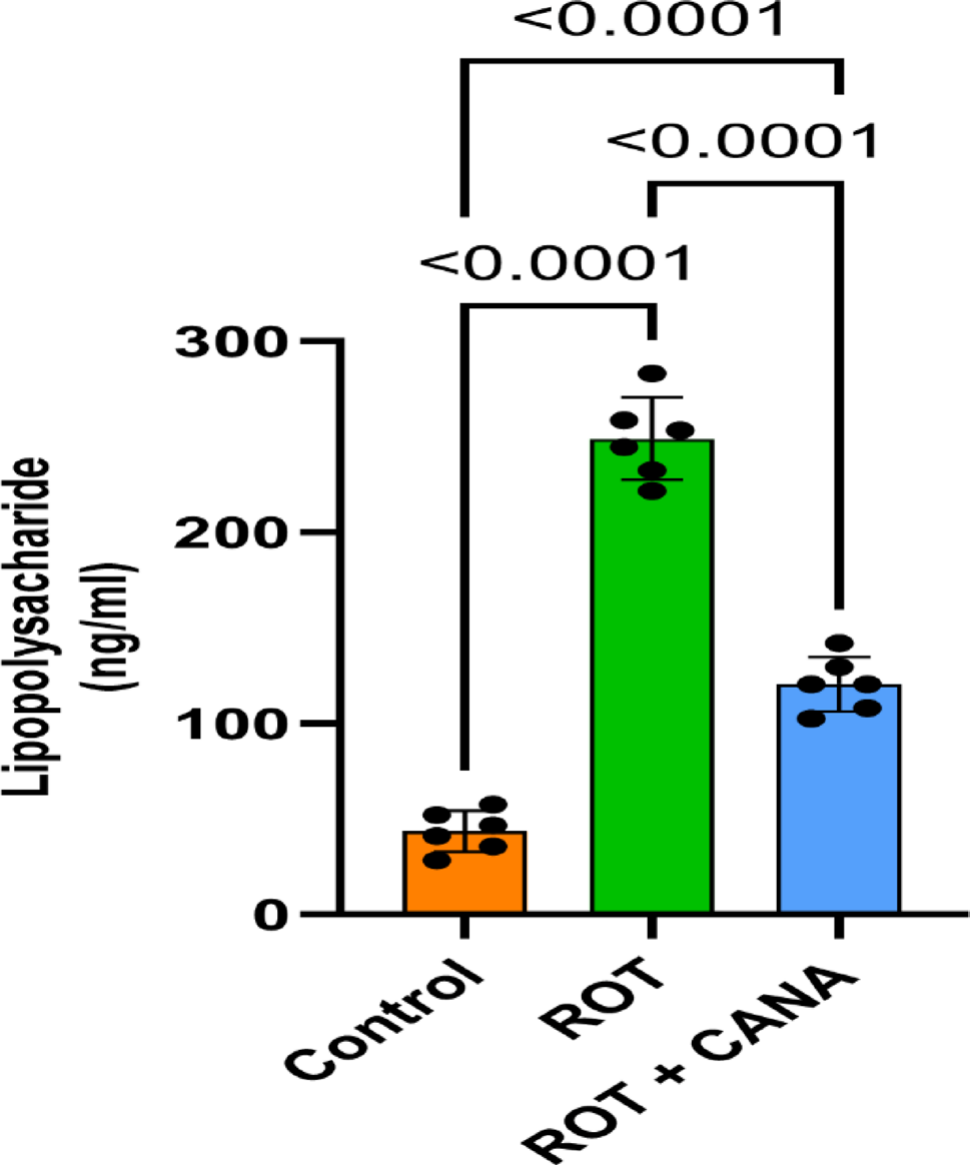
Effect of CANA treatment on LPS serum levels in ROT‑induced PD in rats. Data are presented as mean ± SD (n = 6). Statistical significance was assessed using one-way analysis of variance (ANOVA) followed by Tukey as a post-hoc test. *Rotenone: ROT; Canagliflozin: CANA*

LPS serum levels were identified to assess the effect of each treatment on intestinal barrier function and endotoxemia (Fig. 16). The ROT group exhibited a substantial rise in the serum LPS levels by 5.7-fold as compared to the control group. On the other hand, the CANA group revealed a significant reduction in the serum LPS levels by 2.06-fold relative to that of the ROT group.

**Fig. 17:**
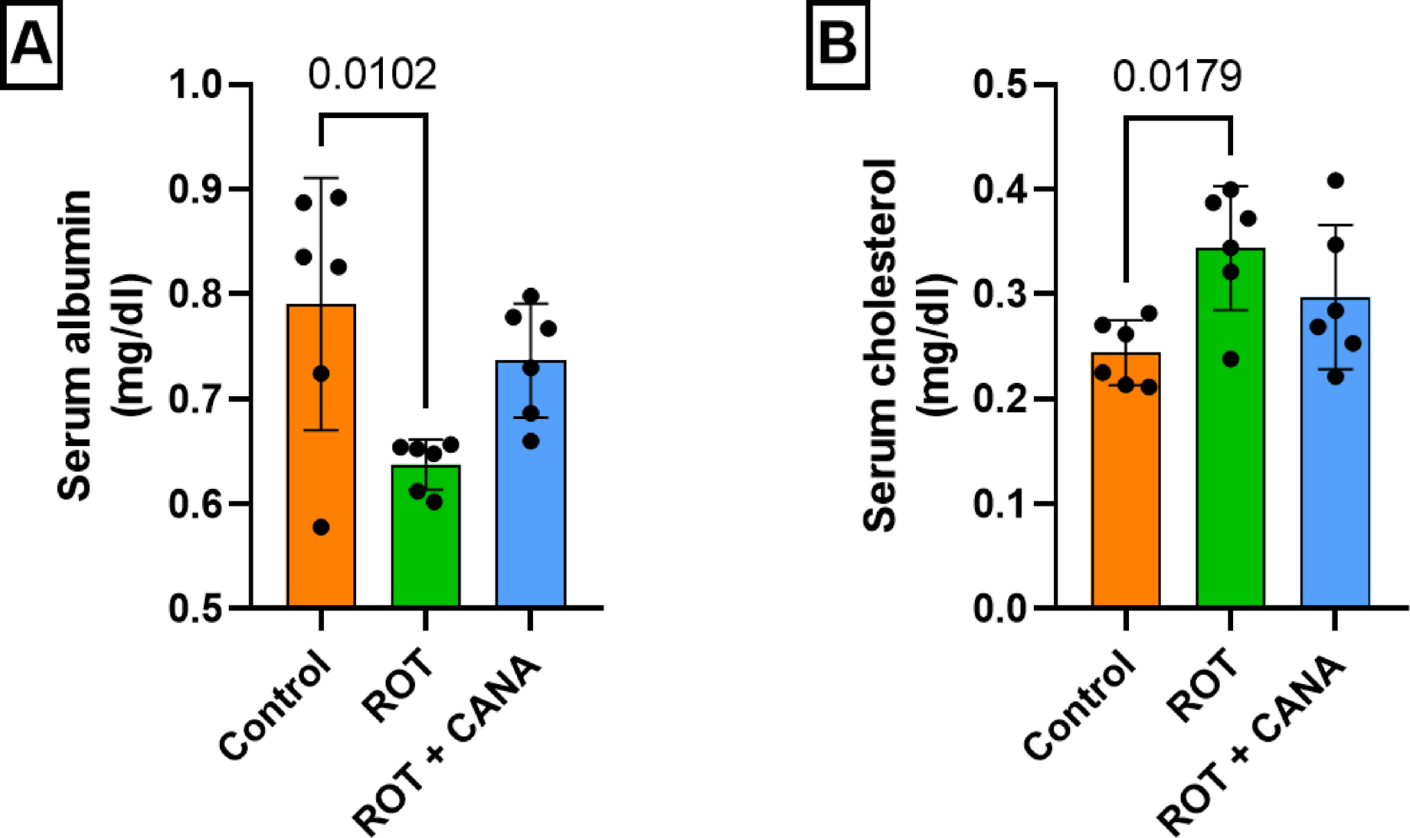
Effect of CANA treatment on albumin (**A**) and cholesterol (**B**) serum levels in ROT‑induced PD in rats. Data are presented as mean ± SD (n = 6). Statistical significance was assessed using one-way analysis of variance (ANOVA) followed by Tukey as a post-hoc test. *Rotenone: ROT; Canagliflozin: CANA*

As illustrated in (Fig. 17A), ROT-treated rats displayed a significant reduction in albumin serum levels relative to the levels of vehicle-treated rats by 1.24-fold. CANA-cotreatment elevated albumin levels; however, it was insignificant. Moreover, ROT intoxication caused a significant elevation in serum cholesterol levels compared to the control group. These changes were reverted, approaching control levels by CANA. Nevertheless, these alterations did not reach statistical significance (Fig. 17B).

### Effect of CANA treatment on oxidative stress and antioxidant capacity ROT‑induced PD in rats

**Fig. 18:**
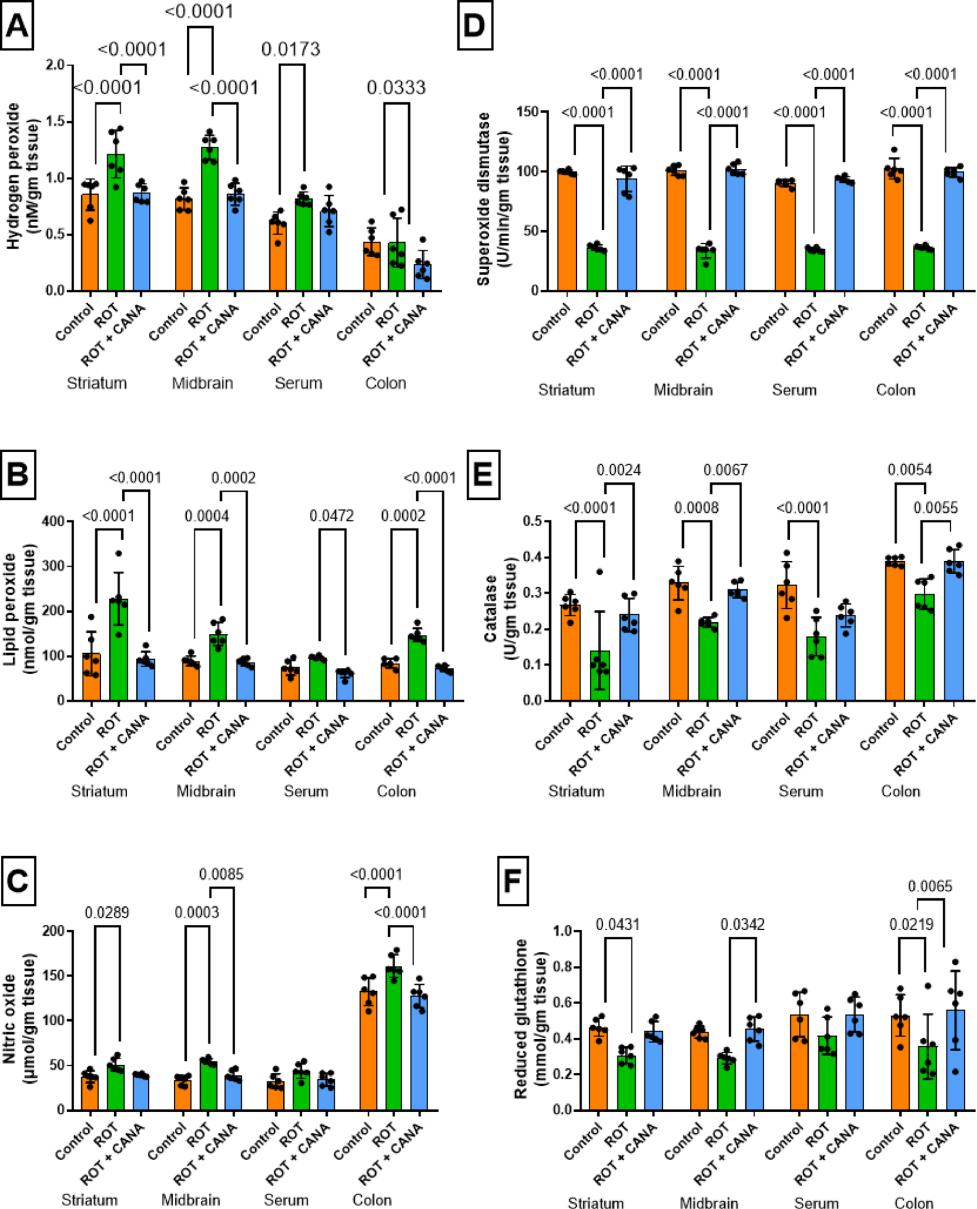
Effect of CANA treatment on the striatal, midbrain, serum and colonic oxidative stress markers; Hydrogen peroxide (**A**), Malondialdehyde (**B**), Nitric oxide (**C**), and the antioxidant markers; Superoxide dismutase (**D**), Catalase (**E**), Reduced glutathione (**F**) in ROT‑induced PD in rats. Data are presented as mean ± SD (n = 6). Statistical significance was assessed using one-way analysis of variance (ANOVA) followed by Tukey as a post-hoc test. *Rotenone: ROT; Canagliflozin: CANA*

The Rotenone-administered group illustrated an elevation in oxidative stress markers, evidenced by a substantial increase in H2O2 striatal, midbrain, and serum levels by 1.42, 1.56, and 1.36 folds, respectively, versus the control group. On the other side, CANA cotreated rats showed a significant lowering of 1.39, 1.48, and 1.84-fold in the striatal, midbrain, and colonic levels of H2O2, respectively (Fig. 18A). Consistently, the Lipid peroxidation level in the ROT group illustrated a marked rise in striatal, midbrain, and colonic tissue when compared to the vehicle group by 2.16, 1.67, and 1.75-fold, respectively. CANA treatment showed a drastic decrease in lipid peroxidation in the striatum, midbrain, serum, and colon by 2.43, 1.72, 1.58, and 2.03, respectively, in comparison with the ROT-treated group (Fig. 18B). In parallel with these findings, ROT substantially enhanced NO levels by 1.35, 1.62, and 1.22-fold in striatum, midbrain, and colon, respectively, compared to those of the control group. Combined treatment with CANA substantially hampered NO striatal, midbrain, and colonic levels by 1.29, 1.4, and 1.26 when compared to the diseased group, while serum levels alterations between different groups showed no significant difference (Fig. 18C).

To investigate the effect of CANA antioxidant defense capacity in ROT‑induced PD in rats, SOD activity was analyzed, and a marked reduction in striatum, midbrain, serum, and colon was observed in ROT-administered rats by 2.74, 2.99, 2.61, and 2.81 folds, respectively, when compared to control rats. CANA cotreatment substantially enhanced SOD activity by 2.58-, 3.03-, 2.69-, and 2.73-fold, respectively, versus the ROT-intoxicated group in the striatum, midbrain, serum, and colon (Fig. 18D). Similarly, disease induction, when compared with control rats, led to a significant decrease in striatal, midbrain, serum, and colonic CAT activity by 1.9, 1.49, 1.79, and 1.31, respectively. CANA treatment reversed these reductions, as evidenced by the notable elevation by 1.71, 1.41, and 1.31 folds in striatal, midbrain, and colonic CAT activity compared with PD-induced rats (Fig. 18E). Further illustration of the ROT-induced redox imbalance, reduced GSH levels displayed a significant decline in striatal and colonic tissue in ROT-treated rats by 1.51 and 1.47 folds, as opposed to the vehicle-treated rats. Rats treated with CANA exhibited a pronounced elevation in midbrain and colonic reduced GSH levels by 1.55 and 1.56 folds as contrasted with the PD group (Fig. 18F).

### Effect of CANA treatment on Inflammasome pathway markers levels ROT‑induced PD in rats

**Fig. 19:**
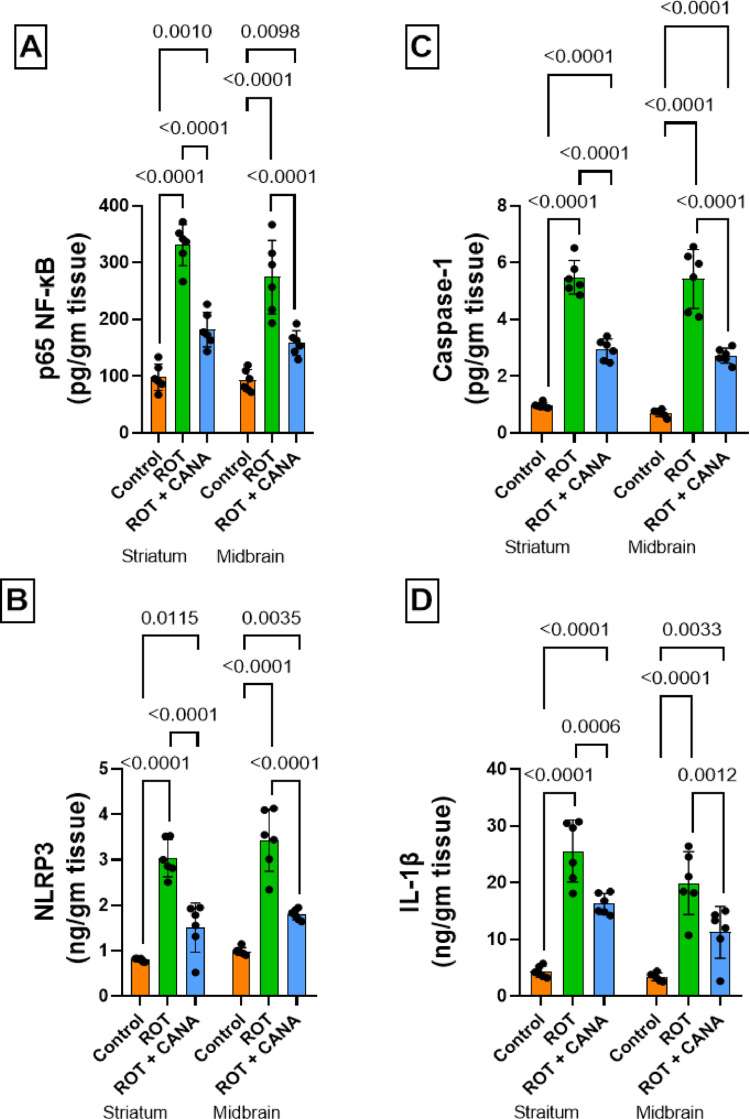
Effect of CANA treatment on striatal and midbrain Inflammasome pathway markers levels; p65 NF-κB (**A**), NLRP3 (**B**), caspase-1 (**C**), and IL-1β (**D**) in ROT‑induced PD in rats. Data are presented as mean ± SD (n = 6). Statistical significance was assessed using one-way analysis of variance (ANOVA) followed by Tukey as a post-hoc test. *Rotenone: ROT; Canagliflozin: CANA.*

As illustrated in Fig. 19, the inflammasome signaling cascade activation was analyzed by assessing p65 NF-κB, NLRP3, caspase-1, and IL-1β levels in the striatum, which were substantially upregulated in PD-induced rats by 3.37, 3.78, 5.67, and 5.92 folds, respectively, while in the midbrain, the elevation was by 2.96, 3.48, 7.65, and 5.86 folds, respectively when set against the untreated group. CANA co-administration inhibited the inflammasome pathway activation, illustrated by a drastic reduction in the levels of p65 NF-κB, NLRP3, caspase-1, and IL-1β in contrast to the disease group, by 1.82, 2.01, 1.86, and 1.57 folds in the striatum, respectively, and by 1.73, 1.91, 1.99, and 1.77 folds in the midbrain, respectively.

## Discussion

The gut microbiota plays a vital role in the development and progression of numerous neurodegenerative diseases, including PD, via mediating the GBA (Li et al. 2023). Emerging data indicate that PD patients suffer from gut dysbiosis, reduced bacterial diversity, and compromised intestinal barrier, which in turn elevates endotoxemia, oxidative stress, and inflammation, inducing α-synuclein aggregation and neurodegeneration (Keshavarzian et al. 2015; Scheperjans et al. 2015; Yang et al. 2024).

The SGLT-2 inhibitor, CANA, has demonstrated antioxidant and anti-inflammatory effects (Dabour et al. 2024, 2025). Moreover, CANA illustrated the modulation and restoration of gut microbiota (He et al. 2024; Lee et al. 2023). Therefore, the present study intended to investigate the possible neuroprotective effect of CANA against ROT-induced PD in rats, focusing on the gut-inflammasome-brain axis.

In order to examine the effect of ROT physiologically and functionally and the safeguarding capacity of CANA, the body weight, blood glucose, and behavioral assessments were analyzed. Consistent with the reported ROT effect on metabolic stress and weight loss mimicking clinical symptoms in PD, ROT administration led to a marked weight loss. The CANA treatment partially attenuated this loss (Johnson and Bobrovskaya 2015). Neither rotenone nor CANA showed notable alterations in glycemic status. Nevertheless, CANA did not cause hypoglycemia, fitting with its SGLT2 inhibition mechanism that only lowers glucose in case of hyperglycemia, while under normoglycemic status maintains glucose level. This equilibrium supports CANA’s metabolic safety in non-diabetic conditions, which was consistent with a previous study (Kuriyama et al. 2014). Regarding motor function, ROT-treated rats exhibited pronounced cataleptic behavior, as well as a substantial decrease in locomotor activity and motor coordination. These findings were consistent with previous research demonstrating the negative effects of ROT treatment on rat motor function (Desouky et al. 2023). Pretreatment with CANA attenuated ROT-induced motor abnormalities, as evidenced by improved locomotor activity, motor coordination in the rotarod test, and reduced cataleptic behavior in the grid and bar tests.

In the ROT-intoxicated group, aligned with previous research, histological examination revealed a predominant neuropathological degeneration in both SN and striatum, marked by severe structural compromise, nuclear loss, and cytoplasmic inclusions (Mohammed et al. 2024). In contrast, tissue integrity was largely restored by CANA treatment in both brain areas.

Moreover, immunohistochemical analysis was done to assess α-synuclein accumulation in the SN and striatum. α-synuclein is necessary for proper synapse function (Yerbury et al. 2016). However, its overexpression and accumulation, due to oxidative stress or inadequate clearance, disrupt cellular homeostasis and trigger neuronal death (Desouky et al. 2023). In harmony with earlier studies (Desouky et al. 2023; Habib et al. 2022), rats intoxicated with ROT demonstrated a substantial spike in α-synuclein accumulation. Collectively, these results support the establishment of PD in this model. Remarkably, the results showed that co-administration of CANA (20 mg/kg) reduced α-synuclein aggregates, indicating its potential neuroprotective potential.

Considering that the gut microbiota and the GBA play an essential role in the pathophysiology of PD (Li et al. 2023), the present study investigated whether ROT-induced neurotoxicity was accompanied by microbial and metabolomic alterations. Several investigations in patients with PD have indicated significant alterations in beta diversity, but no matching changes in alpha diversity, of the gut microbiome (Cirstea et al. 2020; Cosma-Grigorov et al. 2020; Hopfner et al. 2017). In our study, 16S rRNA amplicon sequencing revealed alterations in the microbial composition of the ROT-intoxicated rats’ gut, and differences in beta diversity but no significant modifications in alpha diversity indices, indicating that ROT mainly induced selective taxonomic redistribution rather than general depletion of microbiota richness, which is consistent with previous findings (Fang et al. 2024). At the taxonomic level, *Parabacteroides* and *Ruminococcaceae*, were abundant in the ROT group. This finding is in line with clinical findings, and with an experimental study that correlated the abundance of the aforementioned taxa with the GBA dysfunction, poorer motor outcomes, and compromised gut barrier integrity (Fang et al. 2024). Enriched *Parabacteroides,* from the *Bacteroidia* class, in the ROT group is relevant since it is a Gram-negative bacterial taxon and has a role in the production of inflammation-inducing LPS, which can promote intestinal immune activation, disrupt epithelial barrier integrity, and activate TLR4/NF-κB-related inflammatory signaling. This enrichment is also in harmony with a previous study of PD induced by 6-OHDA injury (Quan et al. 2025). Additionally, echoing previous research on PD patients, the present investigation illustrated that the genus *Prevotella* of the class *Bacteroidia*, which was depleted in the PD model rats, probably reflecting the dysbiosis associated with ROT administration, was partially restored with CANA treatment (except for *Prevotella_9*). *Prevotella* has previously been associated with neuroprotective impact and enhanced intestinal barrier function. since it is well known to ferment dietary fibers and produce SCFAs, such as butyrate and propionate, which lower inflammation and maintain the firmness of tight junctions (Bedarf et al. 2017). Moreover, *Ruminococcaceae* was enriched in the ROT group, and this enrichment is in accordance with previous experimental and clinical studies where *Ruminococcaceae* abundance has been reported to vary in neurodegenerative and inflammatory diseases; it was demonstrated to be higher in PD subjects compared to the healthy controls (Cui et al. 2023; Fang et al. 2024; Shen et al. 2021; Zhang et al. 2022). Furthermore, A ROT model investigation also reported that altered serum amino acids were significantly associated with gut microbiota composition, particularly *Ruminococcaceae* and *Ruminiclostridium*, suggesting a link between this family and host metabolic remodeling (Yan et al. 2022). In this investigation, PD-induced rats showed a higher abundance of *Ruminococcaceae*. *Ruminococcaceae* abundance was also found to be higher in the feces of patients with PD having mild cognitive impairment, compared to those with PD having normal cognition and healthy controls (Ren et al. 2020). Furthermore, its abundance was correlated with the severity and duration of the disease (Hegelmaier et al. 2020; Hill-Burns et al. 2017). Interestingly, a recent study revealed a substantial negative link between *Ruminococcaceae* abundance and intestinal mucosa thickness, and a positive correlation with motor impairments (Fang et al. 2024). CANA co-administration in our study reversed these alterations.

These microbial modulation were paralleled by fecal metabolites alterations, ROT-administered rats revealed an altered fecal metabolome, which could affect pathways related to energy metabolism, neurotransmitter production, and gut microbiota activity. CANA cotreatment appeared to modulate and partially restore the normal fecal metabolome. The partial overlap in PCA clusters, despite distinct PD-related alterations, emphasizes ROT’s small but clinically relevant metabolic abnormalities, which were better represented by the supervised model, OPLS-DA. The CANA group’s associate restoration of key metabolites supports its role in maintaining microbiota-driven metabolic balance, which is consistent with its effects on gut microbial composition (Zeng et al. 2024).

The metabolomic profiling of fecal samples in this study provides compelling evidence for the role of the gut-inflammasome–brain axis in the pathogenesis of Parkinson’s Disease (PD), as well as the mechanistic efficacy of the administered treatment. The distinct separation of groups in the OPLS-DA models, driven by highly specific VIP-ranked metabolites, illustrates a clear transition from a pro-inflammatory, dysbiotic gut environment to a neuroprotective, homeostatic state. The pathological fingerprint of the PD group was primarily defined by the accumulation of ribitol and the concurrent depletion of 2-hydroxy-2-methylbutanoic acid. Ribitol, a pentose alcohol, serves as a biomarker of carbohydrate fermentation, signaling a profound state of gut dysbiosis. Elevated ribitol levels have been observed in the cerebrospinal fluid (CSF) of patients with Parkinson’s disease (Liao et al. 2025). This microbial shift likely increases intestinal barrier permeability, allowing systemic translocation of pro-inflammatory triggers. 2-Hydroxy-2-methylbutanoic acid is a key intermediate in the microbial metabolism of branched-chain amino acids (Zhou et al. 2025). Its significant reduction in the PD group compared to the control group suggests disruption of microbial protein fermentation, an imbalance that significantly contributes to intestinal dysbiosis. Interestingly, while this metabolite was depleted in the PD state compared to healthy controls, it remained low in the treated group. This suggests that the therapeutic intervention does not merely attempt to revert the gut to a baseline but rather redirects metabolic resources toward more critical protective pathways, such as the restoration of the intestinal lipidome.

Simultaneously, the increased fecal levels of myo-inositol, a well-established marker of astrogliosis and microglial activation (Rae 2014), indicate systemic priming of the neuroinflammatory response. This aligns with the striatal activation of the NLRP3 inflammasome identified in histological examination, suggesting that alterations in the fecal metabolome may serve as a reliable peripheral indicator of central glial stress and neuroinflammatory activity. This correlates directly with our observations of elevated striatal IL-1β and p65 NF-κB in the PD group. The marked suppression of myo-inositol in the treated group (F-C) indicates that the therapeutic intervention successfully attenuated this systemic inflammatory cascade.

A cornerstone effect of CANA was the significant enrichment of (*Z*,*Z*)-9,12-octadecadienoic acid (linoleic acid) and long-chain alkanes (n-heptadecane and n-heneicosane). Linoleic acid, an essential polyunsaturated fatty acid (PUFA), is highly susceptible to degradation by reactive oxygen species (ROS). Its profound depletion in the untreated PD group serves as a metabolic footprint of lipid peroxidation and systemic oxidative stress (Vesga-Jiménez et al. 2022). The restoration of linoleic acid following CANA treatment demonstrates a potent antioxidant effect, protecting essential fatty acids from oxidative damage and preserving the structural integrity of the intestinal membrane (Xicoy et al. 2019). Besides, the metabolic profile of CANA-treated group (F-C) was characterized by elevated levels of long-chain alkanes, including n-heptadecane and n-heneicosane. In fecal metabolomics, enrichment of such hydrocarbons may reflect restoration of microbial lipid metabolism and/or metabolic signatures associated with therapeutic intervention.

Interestingly, the PD group (F-B) exhibited high levels of fatty acid amides (FAAs), myristamide, N-methyl; and arachidamide, N-methyl. While these FAAs possess anti-inflammatory properties, their upregulation in the diseased state often represents a reactive compensatory mechanism (Pacher and Kunos 2013). This suggests that the host may be attempting to suppress NF-κB-mediated inflammasome pathway as a compensatory anti-inflammatory response. However, this endogenous mechanism appears insufficient to effectively prevent or attenuate disease progression in the untreated group. The CANA cotreatment appears to restore metabolic homeostasis by shifting the gut environment from an “active combat” state characterized by reactive lipid signaling toward a more balanced and regulated metabolic profile.

In conclusion, the integration of these metabolomic findings suggests that CANA cotreatment mitigates PD pathogenesis and restores key metabolites supporting its role in maintaining microbiota-driven metabolic balance, which is consistent with its effects on gut microbial composition, and function (Zeng et al. 2024). The marked reduction in ribitol indicates a successful suppression of gut dysbiosis, whereas the increased abundance of long-chain alkanes and the concurrent recovery of linoleic acid highlight the restoration of healthy microbial lipid metabolism and the quenching of lipid peroxidation. Collectively, these shifts imply that the therapeutic intervention not only restores intestinal metabolic integrity but also actively inhibits gut-derived inflammatory signaling, thereby neutralizing the systemic processes involved in striatal NLRP3 inflammasome activation.

Such microbiota and metabolites disturbances were accompanied by intestinal dysfunction, resulting in elevated colonic oxidative stress, which was hampered by CANA treatment. These biochemical changes were in alignment with intestinal histopathological alterations, which revealed altered mucosal architecture, ulceration, and edema. Interestingly, CANA co-administration preserved normal colonic architecture and mucosal layer. ROT alteration of colonic architecture was accompanied by disturbances in the intestinal integrity and enhanced gut permeability, as supported by reduced expression of the tight junction protein claudin-1 that regulates paracellular ion and solute transport while preserving epithelial cohesion. When tight-junction function is interrupted, paracellular permeability increases, allowing bacterial toxins to get through the barrier (Edens and Parkos 2000), enabling the translocation of the endotoxin LPS to the systemic circulation, as shown by elevated serum LPS levels in the ROT group, representing endotoxemia.

Elevated systemic LPS levels may represent a link between peripheral dysbiosis and central neuroinflammation. Endotoxemia was associated with heightened systemic oxidative stress, illustrated by increased lipid peroxidation and enhanced levels of H2O2 and NO and at the same time disrupted antioxidant defense by decreased SOD, CAT and reduced GSH levels that was in line with previous investigations (Ishola et al. 2022; Yang et al. 2024). In contrast, CANA co-therapy was associated with partial normalization of gut dysbiosis, maintaining claudin-1 expression, modulating the gut permeability, resulting in decreased LPS translocation, which consequently prevented the propagation of systemic oxidative stress. In the ROT group, such peripheral endotoxemia and oxidative stress may further compromise BBB integrity, facilitating entry of inflammatory cytokines and oxidative products into the CNS (Liang et al. 2025).

In parallel, ROT-treated rats showed increased oxidative stress within the brain, as evidenced by increased oxidative stress markers and decreased antioxidant defense in the midbrain and striatum, along with neuroinflammation in the CNS (Catorce and Gevorkian 2016; Chen et al. 2008). LPS is known to stimulate the NF-κB/NLRP3 inflammasome axis, which primes the NLRP3 and pro-inflammatory cytokines (pro-IL-1β). NLRP3 then forms the inflammasome, which activates caspase-1 and converts cytokines into their active forms, leading to inflammation and pyroptotic cell death (Elhawary et al. 2025; Zakaria et al. 2025). Dysregulation of this pathway is connected with neuroinflammatory and neurodegenerative disorders (Voet et al. 2019). This was demonstrated in our study, where the inflammasome pathway was upregulated in the striatum and midbrain of the ROT-intoxicated rats. On the other hand, CANA treatment attenuated the neuroinflammation as illustrated by reduced inflammasome pathway marker.

Some limitations to the current investigation should be acknowledged. First, the lack of a CANA-only control group makes it difficult to separate treatment-specific baseline effects from protective actions during pathological settings, despite earlier reports indicating that CANA causes minor physiological changes under normoglycemic conditions. Second, while the current data indicate the participation of linked gut, inflammatory, and brain pathways, direct mechanistic validation is restricted because no selective pathway blockage or targeted microbial functional experiments were conducted. Thus, causal links between microbial changes, endotoxemia, inflammasome activation, and neuroprotection should be evaluated with caution.

Translationally, these findings are significant because CANA is already approved clinically, which may facilitate future medication repurposing protocols if neuroprotective efficacy is verified in additional mechanistic and clinical studies. However, the discrepancies between ROT-induced toxicity and human disease progression must be carefully interpreted before applying them to clinical PD. Specifically, ROT-induced PD-like pathology remains a toxicant-based model that reproduces some PD-relevant features, such as mitochondrial dysfunction, oxidative injury, neuroinflammation, α-synuclein accumulation, nigrostriatal pathology, and motor impairment. However, it does not fully capture the multifactorial and progressive nature of idiopathic human PD. Therefore, validation in additional models, including α-synuclein-based and genetic PD models, would strengthen translational relevance. Furthermore, while CANA is clinically licensed for metabolic disorders, repurposing it for non-diabetic PD patients would necessitate a thorough assessment of dose translation, pharmacokinetics, and safety.

In conclusion, for the first time, this research sheds light on the potential neuroprotective effect of CANA in a rat model of ROT-induced PD. CANA administration was associated with modulation of the gut microbiota, improved intestinal integrity, reduced systemic endotoxemia, oxidative stress, and inflammasome-associated signaling, and attenuated α-synuclein aggregation. Taken together, these results suggest that CANA could be a potential therapeutic option through multi-target modulation of interconnected peripheral and central pathological processes, offering an integrated approach to alleviate PD neurodegeneration.

## Supplementary Information

Below is the link to the electronic supplementary material.

Supplementary file 1

Supplementary file 2

## Data Availability

Data will be made available upon request from the corresponding author. Sequence datasets generated for this study are deposited at NCBI sequence reads archive (SRA) under project number #PRJNA1415681.
