# Supplementary figures and images for: Canagliflozin attenuates Parkinson’s disease and is associated with modulation of gut-inflammasome–brain axis in rats
Source: Inflammopharmacology. 2026 Jun 22;34(7):4753–82. doi: 10.1007/s10787-026-02292-5 (PMC13391799; doi:10.1007/s10787-026-02292-5)

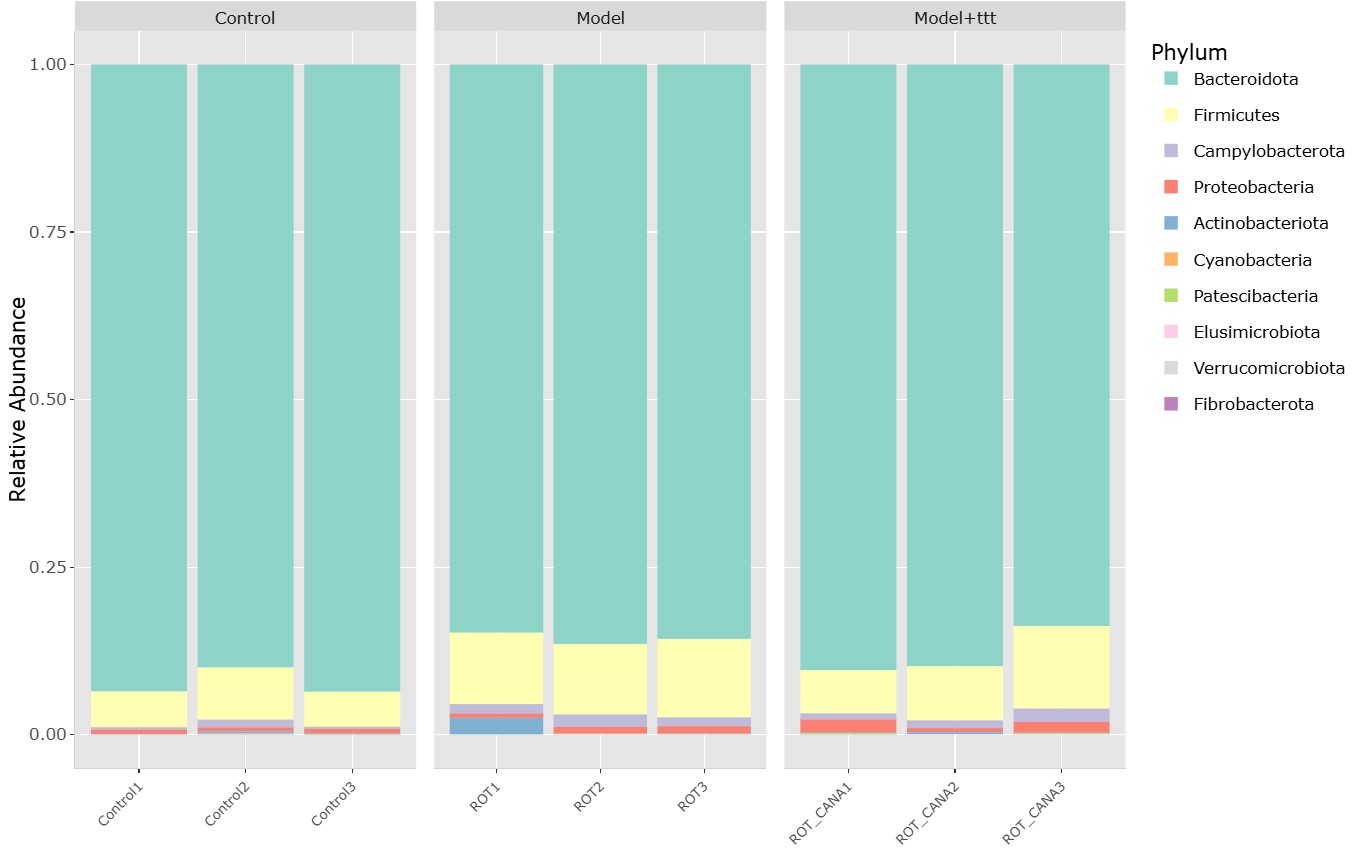

Supplement: Supplementary file 1 [file 10787_2026_2292_MOESM1_ESM.jpg]

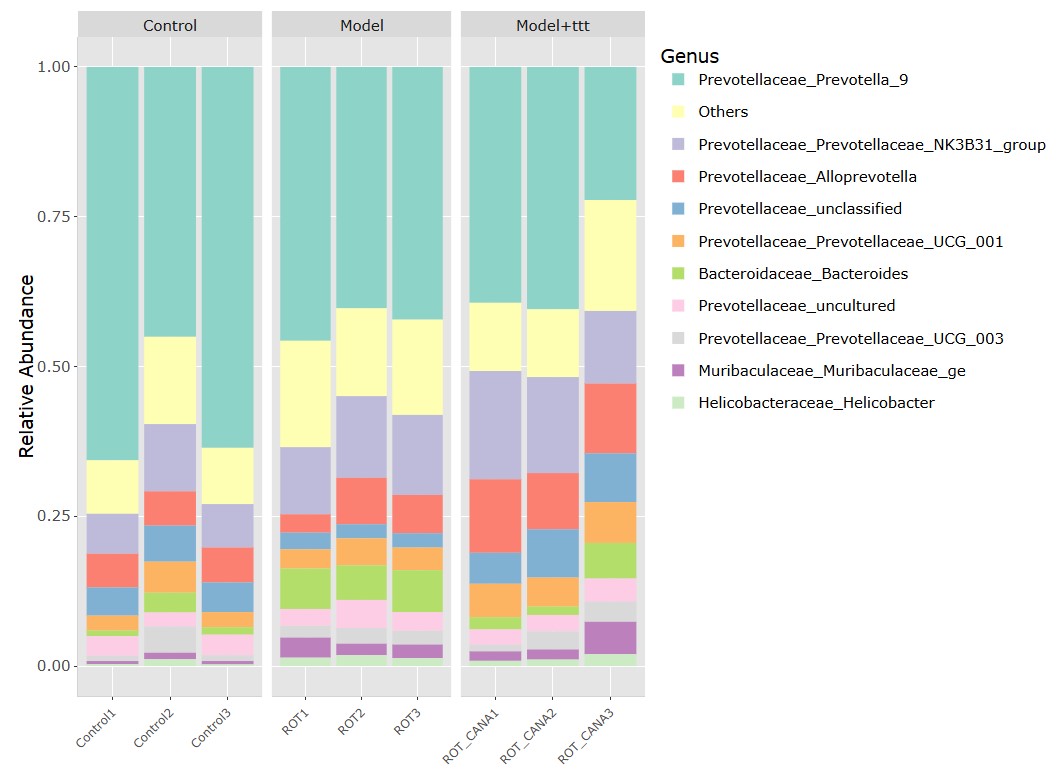

Supplement: Supplementary file 2 [file 10787_2026_2292_MOESM2_ESM.jpg]
